# Productivity-driven decoupling of microbial carbon use efficiency and respiration across global soils

**DOI:** 10.1126/sciadv.adz5319

**Published:** 2026-01-14

**Authors:** Yongxing Cui, Shushi Peng, Manuel Delgado-Baquerizo, Daryl L. Moorhead, Robert L. Sinsabaugh, César Terrer, Thomas P. Smith, Yakov Kuzyakov, Josep Peñuelas, Biao Zhu, Feng Tao, Songbai Hong, Ji Chen, Matthias C. Rillig

**Affiliations:** ^1^ Institute of Biology, Freie Universität Berlin, Berlin 14195, Germany.; ^2^ Sino-French Institute for Earth System Science, College of Urban and Environmental Sciences, Peking University, Beijing 100871, China.; ^3^ Laboratorio de Biodiversidad y Funcionamiento Ecosistémico, Instituto de Recursos Naturales y Agrobiología de Sevilla (IRNAS), CSIC, Av. Reina Mercedes 10, E-41012 Sevilla, Spain.; ^4^ Department of Environmental Sciences, University of Toledo, Toledo, OH 43606, USA.; ^5^ Department of Biology, University of New Mexico, Albuquerque, NM 87131, USA.; ^6^ Department of Civil and Environmental Engineering, Massachusetts Institute of Technology, Boston, MA 02139, USA.; ^7^ Department of Life Sciences, Imperial College London, Silwood Park Campus, Ascot, Berkshire SL5 7PY, UK.; ^8^ Department of Agricultural Soil Science, Department of Soil Science of Temperate Ecosystems, University of Goettingen, Goettingen 37077, Germany.; ^9^ Peoples Friendship University of Russia RUDN University, Moscow 117198, Russia.; ^10^ Global Ecology Unit CREAF-CSIC-UAB, CSIC, Bellaterra, Catalonia 08913, Spain.; ^11^ CREAF, Cerdanyola del Vallès, Catalonia 08913, Spain.; ^12^ State Key Laboratory for Vegetation Structure, Function and Construction (VegLab), Ministry of Education Key Laboratory for Earth Surface Processes, and College of Urban and Environmental Sciences, Peking University, Beijing, China.; ^13^ Department of Ecology & Evolutionary Biology, Cornell University, Ithaca, NY 14853, USA.; ^14^ School of Urban Planning and Design, Shenzhen Graduate School, Peking University, Shenzhen 518055, China.; ^15^ State Key Laboratory of Loess Science, Institute of Earth Environment, Chinese Academy of Sciences, Xi’an 710061, China.

## Abstract

Despite extensive research on soil microbial carbon (C) use efficiency (CUE), its linkage to actual soil C storage remains ambiguous. A key uncertainty is that CUE estimates from short-term labeling incubations assume a linear negative relationship with respiration rates, overlooking nonlinear interactions and long-term microbial acclimation. Here, we use a stoichiometry-based approach to estimate CUE (CUE_ST_), which links soil resource availability to microbial demand and captures microbial adaptability under resource constraints. We synthesized 1094 paired observations of CUE_ST_ and heterotrophic respiration rate (*R*_h_) across natural ecosystems and found a nonlinear relationship between them governed by ecosystem productivity. In low-productivity arid and cold regions, CUE_ST_ declined with increasing *R*_h_, whereas in productive tropical and temperate regions, CUE_ST_ stabilized at a low level (0.27 ± 0.11) as *R*_h_ exceeded 340 ± 10.8 grams of C per square meter per year. This shift reflects microbial trade-offs between C assimilation and stoichiometric homeostasis, revealing a decoupling of microbial growth from respiration that limits the capacity of productive ecosystems to store additional soil C.

## Teaser

Resource-dependent microbial adaptability decouples microbial growth from respiration and limits soil carbon storage.

## INTRODUCTION

Soil microbial carbon (C) assimilation and heterotrophic respiration (*R*_h_) are two basic microbial metabolic processes that collectively control organic C retention in soils (*1*–*3*). When the total microbial C uptake remains constant, higher assimilation for growth combined with lower *R*_h_ indicates more efficient biomass production, which enhances soil organic C retention (*4*–*6*). Culture-based studies, from strains to community levels, have shown that microbial growth efficiency is more stable than *R*_h_ under changing environmental conditions (*7*, *8*). Recent global assessments further suggest that warming accelerates *R*_h_ but does not have a clear or consistent effect on microbial growth (*9*, *10*).

These differential responses imply a decoupling between microbial C assimilation efficiency, commonly described as microbial C use efficiency (CUE), and *R*_h_ under specific environmental constraints. This decoupling challenges the long-held assumption that CUE uniformly declines with increasing *R*_h_, even though CUE definitions vary among measurement approaches (*2*). It may also represent a key source of uncertainty in linking CUE to soil C storage and dynamics (*6*, *11*, *12*). Under nutrient-limited conditions, particularly low nitrogen (N) and phosphorus (P) availability, microorganisms may sustain growth by investing in energetically expensive enzyme production and efficient nutrient recycling, which increases C efflux while maintaining biomass production, thereby decoupling CUE from *R*_h_ (*13*, *14*). Nevertheless, this potential decoupling has been neither empirically tested across natural ecosystems nor understood in terms of the potential mechanisms involved.

This knowledge gap is likely attributable to three main reasons. First, most existing studies assume a linear negative relationship between CUE and *R*_h_ because widely used approaches, such as those based on ^13^C/^14^C-labeled substrates and ^18^O-labeled water, calculate CUE from pulse *R*_h_ and thus inherently generate a negative correlation between the two (*15*–*17*). However, this approach overlooks mounting evidence for widespread nonlinear relationships in soil C cycling across ecosystems (*7*, *18*). Second, *R*_h_ measurements used for estimating CUE are generally conducted on disturbed soils under short-term laboratory incubations, which reflect immediate metabolic responses to added substrates, rather than long-term microbial adaptability (*3*, *17*, *19*). Moreover, changes in the availability of one resource (e.g., C) can trigger cascading metabolic responses to other resources through priming effects and nutrient mining (*20*–*22*). These processes could obscure trade-offs between microbial C assimilation and the maintenance of stoichiometric homeostasis in natural ecosystems (*23*). Third, there is a lack of simultaneous, independent measurements of CUE and *R*_h_ across a range of natural ecosystems, limiting our ability to assess their relationship across environmental gradients.

**Fig. 1.**
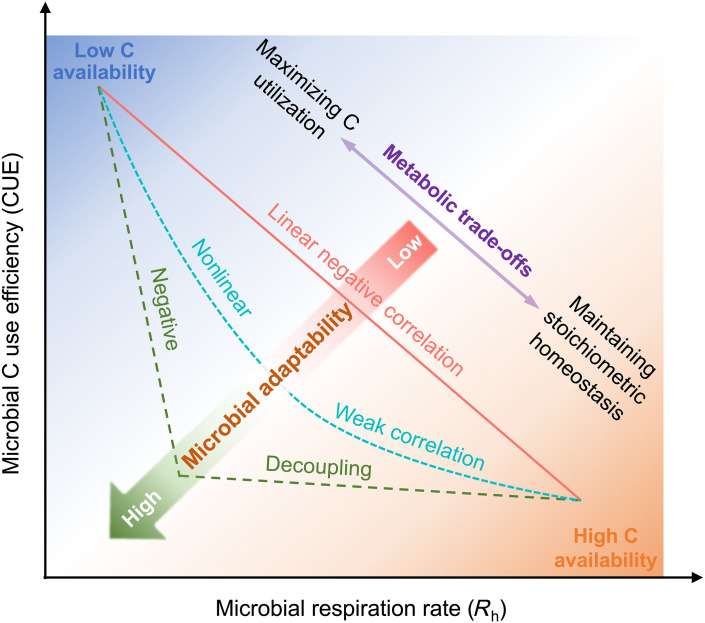
Conceptual framework illustrating the possible relationships between microbial CUE and *R*_h_ on the basis of stoichiometric theory and microbial community theory. The stoichiometric theory suggests a linear negative relationship between CUE and *R*_h_, as microorganisms respire excess C, e.g., via overflow respiration to maintain their elemental stoichiometric homeostasis, thereby reducing CUE as *R*_h_ increases (*4*, *69*). However, the relationship between CUE and *R*_h_ may become nonlinear or even decoupled (i.e., no correlation) with increasing resource availability and microbial physiological adaptation. Microbial adaptation tends to stabilize CUE, whereas increasing resource availability, particularly C, can disproportionately elevate *R*_h_ (*33*, *70*–*72*). Note that the specific meaning of CUE depends on the approaches used for its determination; here, it generally refers to the C assimilation efficiency of the microbial community.

**Fig. 2.**
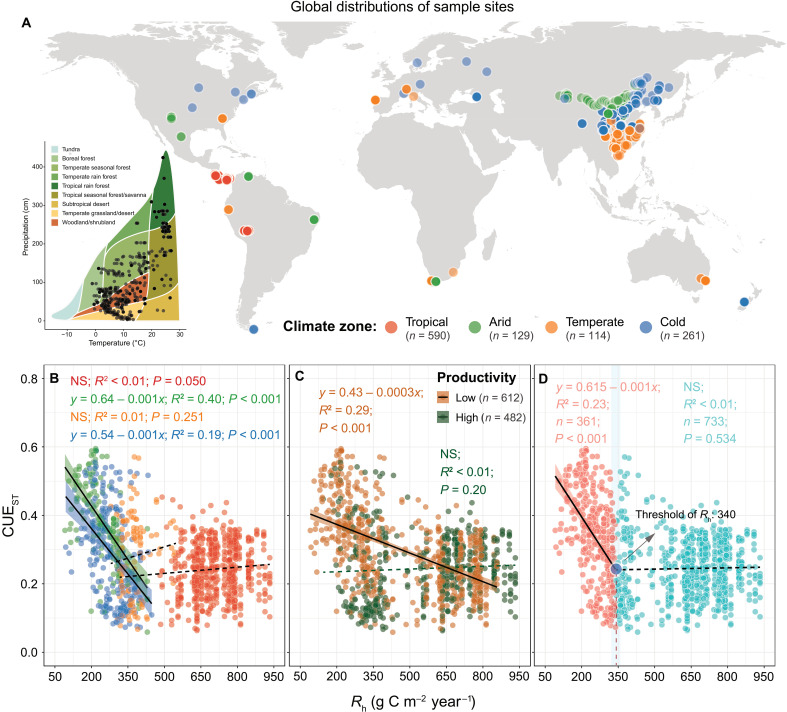
Geographic distributions of sample sites and relationships between CUE_ST_ and average annual *R*_**h**_. (**A**) A total of 1094 observations at 447 sites from 160 studies were collected for estimating CUE_ST_ using a stoichiometric-based method and are distributed across nearly all Whittaker biomes and major climatic zones. (**B** and **C**) Relationships between CUE_ST_ and *R*_h_ among climate zones (B) as well as in low-productivity (LAI < 3) and high-productivity (LAI > 3) ecosystems (C) were identified using generalized linear models. The shaded area is the 97.5% confidence interval of the linear regressions. All continuous lines are significant at *P* < 0.05, whereas dashed lines are not significant (NS; *P* > 0.05). (**D**) CUE_ST_ decoupled from *R*_h_ when *R*_h_ > 340 ± 10.8 g C m^−2^ year^−1^. The threshold of *R*_h_ was estimated using piecewise regression analyses (see table S5 for detailed results), and the relationships between CUE_ST_ and *R*_h_ before and after this threshold were identified using generalized linear models. The blue circle indicates the threshold of *R*_h_, and the shaded area is the 97.5% confidence interval of the threshold. The solid black lines indicate model fits between CUE_ST_ and *R*_h_ (*P* < 0.05).

To explore the potential decoupling between CUE and *R*_h_, we compiled a global dataset of 1094 paired, independently derived observations of CUE and *R*_h_ across natural ecosystems. Specifically, we estimated CUE using a culture-independent stoichiometric model (*24*), which accounts for microbial enzyme allocation strategies to minimize elemental imbalances between soil resource supply and microbial growth demand. The estimated CUE (“CUE_ST_” hereafter) reflects in situ microbial traits, with higher values indicating more efficient C utilization relative to nutrient acquisition. To obtain corresponding *R*_h_ values, we matched the geographic coordinates (latitude and longitude) of each CUE_ST_ observation with average annual *R*_h_ (“*R*_h_” hereafter) from the latest global Soil Respiration Database (*25*), which characterizes long-term patterns of microbial respiration. We hypothesize that CUE_ST_ is negatively correlated with *R*_h_ under low C availability (e.g., low plant-derived C inputs), as microorganisms encounter a trade-off between C assimilation and respiratory loss. However, CUE_ST_ and *R*_h_ could decouple under low nutrient conditions because of increased C expenditure to acquire and recycle limiting nutrients, particularly N and P, to maintain stoichiometric homeostasis (Fig. 1). To test this hypothesis, we examined the relationship between CUE_ST_ and *R*_h_ across global ecosystems (Fig. 2A) and evaluated the environmental drivers with nine variables representing temperature, water, C, and nutrient availability.

## RESULTS

### Relationships between CUE and *R*_h_ at a global scale

**Fig. 3.**
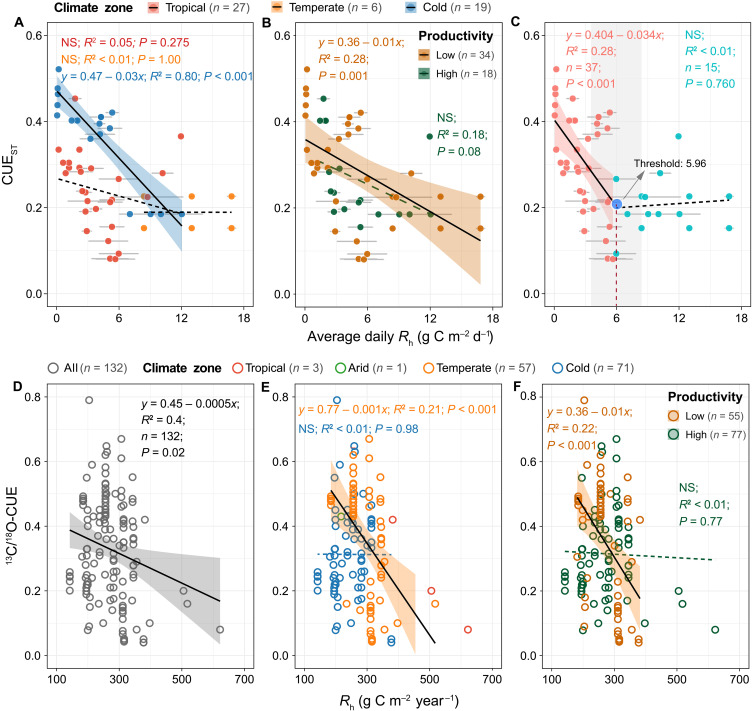
Relationships between CUE_ST_ and average daily *R*_h_ or between ^13^C/^18^O-CUE and average annual *R*_h_ across global soils. (**A** and **B**) Relationships between CUE_ST_ and average daily *R*_h_ among climatic zones (or in low- and high-productivity ecosystems) were identified using generalized linear models. The shaded area is the 97.5% confidence interval of the linear regressions. All continuous lines are significant at *P* < 0.05, whereas dashed lines are not significant (*P* > 0.05). (**C**) CUE_ST_ decoupled with average daily *R*_h_ when the average daily *R*_h_ was larger than the threshold of 5.96 ± 1.44 g C m^−2^ day^−1^. The threshold of average daily *R*_h_ was estimated using piecewise regression analyses (see table S6 for detailed results), and the relationships between CUE_ST_ and average daily *R*_h_ before and after this threshold of average daily *R*_h_ were identified using generalized linear models. The blue circle indicates the threshold of average daily *R*_h_, and the shaded area is the 97.5% confidence interval of the threshold. The solid black lines indicate model fits between CUE_ST_ and average daily *R*_h_ (*P* < 0.05). In (A) to (C), the gray line through each data point represents the standard deviation of the mean of average daily *R*_h_. (**D** to **F**) Relationships between ^13^C/^18^O-CUE and *R*_h_ among climatic zones (or in low- and high-productivity ecosystems) were identified using generalized linear models. Note that (i) no matching data of CUE_ST_ with average daily *R*_h_ were collected in the arid zone; (ii) we also did not fit the relationships between ^13^C/^18^O-CUE and *R*_h_ for the tropical and arid zones in (E) because of the small number of observations, (iii) no threshold of *R*_h_ was identified in the relationship between ^13^C/^18^O-CUE and *R*_h_ using piecewise regression analyses.

We found that the relationship between CUE_ST_ and *R*_h_ varied across climatic zones and productivity levels. In arid and cold zones, CUE_ST_ declined significantly with increasing *R*_h_ (*P* < 0.001; Fig. 2B), whereas in tropical and temperate zones, it remained stable at a relatively low level (0.27 ± 0.11; *P* > 0.05). Because the shift in CUE_ST_-*R*_h_ relationships across climate zones appeared to be strongly linked to ecosystem productivity, we categorized sites into low-productivity [leaf area index (LAI) < 3] and high-productivity (LAI > 3) ecosystems on the basis of the observed LAI range of 0.06 to 5.69 across sites. In low-productivity ecosystems, CUE_ST_ was negatively correlated with *R*_h_ (*n* = 612, *R*^2^ = 0.29, *P* < 0.001; Fig. 2C), whereas this relationship was absent in high-productivity ecosystems (*n* = 482, *R*^2^ < 0.01, *P* = 0.20). We further identified a global threshold of *R*_h_ at 340 ± 10.8 g C m^−2^ year^−1^ (Fig. 3D and table S5). Below this threshold, CUE_ST_ declined significantly with increasing *R*_h_ (*n* = 361, *R*^2^ = 0.23, *P* < 0.001); above it, no significant correlation was detected (*n* = 733, *R*^2^ < 0.01, *P* = 0.53).

Further support for decoupling between CUE and *R*_h_ was provided by two additional independent datasets: average daily *R*_h_ (*n* = 52) and ^13^C/^18^O-measured CUE (*n* = 132) (Fig. 3 and fig. S7). First, CUE_ST_ was uncorrelated with average daily *R*_h_ in tropical and temperate zones (*P* > 0.05; Fig. 3A) but decreased significantly with average daily *R*_h_ in the cold zone (*R*^2^ = 0.80, *P* < 0.001). Along a productivity gradient, a negative relationship was observed in low-productivity ecosystems (*R*^2^ = 0.28, *P* = 0.001; Fig. 3B), whereas no significant correlation was found in high-productivity ones (*P* = 0.08). Across all sites, we identified a threshold of average daily *R*_h_ at 6.0 ± 1.4 g C m^−2^ day^−1^ (Fig. 3C and table S7): Below this threshold, CUE_ST_ declined significantly with average daily *R*_h_ (*R*^2^ = 0.28, *P* < 0.001); above it, the relationship was not significant (*P* = 0.76). Note that this daily *R*_h_ threshold (equivalent to ~2190 g C m^−2^ year^−1^ when multiplied by 365 days) is about 6.4 times higher than the annual *R*_h_ threshold described above (340 g C m^−2^ year^−1^). This discrepancy is expected given that daily rates are usually measured during the growing season, whereas average annual values integrate fluxes across the entire year. Second, using the ^13^C/^18^O-CUE dataset, we found a weak but significant negative correlation of ^13^C/^18^O-CUE with *R*_h_ across all sites (*P* = 0.02; Fig. 3D). Regionally, ^13^C/^18^O-CUE declined with *R*_h_ in the temperate zone (*R*^2^ = 0.21, *P* < 0.001; Fig. 3E) but was decoupled in the cold zone (*P* = 0.98). A negative relationship was also observed in low-productivity ecosystems (*R*^2^ = 0.22, *P* < 0.001; Fig. 3F), whereas no significant relationship was found in high-productivity ecosystems (*P* = 0.77).

### Environmental drivers of the relationships between CUE and *R*_h_

**Fig. 4.**
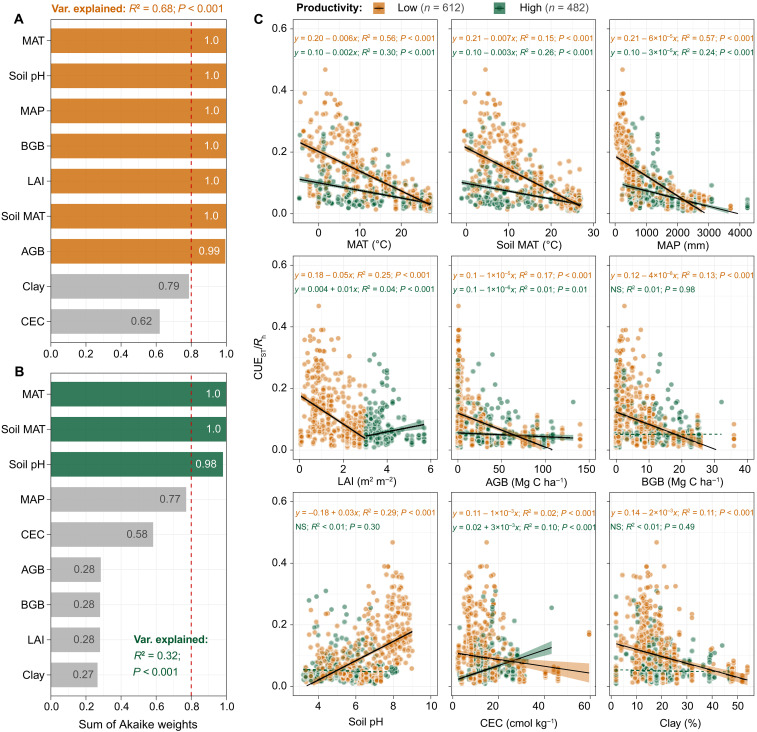
Effects of environmental factors on the relative change of CUE_ST_ versus average annual *R*_h_ in low- and high-productivity ecosystems. (**A** and **B**) The analysis of mixed-effects model selection was used to evaluate the relative importance of these variables affecting CUE_ST_/*R*_h_. Values of the sum of Akaike weights were estimated on the basis of corrected Akaike’s information criteria. A threshold value of 0.8 (red dashed line) was set to identify the most important variables. (**C**) The effects of these environmental variables on CUE_ST_/*R*_h_ in low- and high-productivity ecosystems were also evaluated using generalized linear models. The shaded area is the 97.5% confidence interval of the linear regressions. All continuous lines are significant at *P* < 0.05, whereas dashed lines are not significant (*P* > 0.05). The relative change of CUE_ST_ versus *R*_h_ is expressed by the CUE_ST_:*R*_h_ ratio (CUE_ST_/*R*_h_). Clay, soil clay content.

We used nine variables that represent the four fundamental controls (i.e., temperature, water, C, and nutrients) for microbial metabolism to further explore the environmental drivers underlying the relationship between CUE and *R*_h_ (Fig. 4). We expressed this relationship as the ratio of CUE_ST_ to *R*_h_ (hereafter “CUE_ST_/*R*_h_”), which reflects their relative changes across environmental gradients. A mixed-effects model selection analysis identified that these nine variables explain 68% of the variation in CUE_ST_/*R*_h_ in low-productivity ecosystems, with seven of the nine variables, spanning all four basic controls, exerting significant effects (*P* < 0.001; Fig. 4A). In contrast, the nine variables explained only 32% of the variation in high-productivity ecosystems, with only three (related to temperature and nutrients) showing significant effects (*P* < 0.001; Fig. 4B). Generalized linear models further confirmed that nearly all nine variables had stronger effects (i.e., higher *R*^2^ values) on CUE_ST_/*R*_h_ in low-productivity ecosystems compared to high-productivity ones (Fig. 4C). These results suggest that in low-productivity ecosystems, CUE_ST_-*R*_h_ relationships are shaped by a broad suite of environmental factors, whereas in high-productivity ecosystems, they are primarily governed by temperature and nutrient availability.

To better understand these patterns across climate zones, we focused on four key indicators [i.e., mean annual soil temperature (soil MAT), mean annual precipitation (MAP), LAI, and soil pH], each representing one of the four basic controls. These factors varied significantly across climatic zones (*P* < 0.001; fig. S11). Among them, soil pH exerted the strongest influence on CUE_ST_/*R*_h_ in tropical and temperate zones, while MAP and LAI were the most influential in arid and cold zones, respectively (fig. S12). Furthermore, multiple statistical analyses consistently showed that LAI, soil MAT, and MAP were the lowest in arid and cold zones, where they had strong negative effects on CUE_ST_ but strong positive effects on *R*_h_ (figs. S13 to S15). These contrasting influences likely contributed to the negative CUE_ST_-*R*_h_ relationships observed in these zones (fig. S16). In contrast, in tropical and temperate zones, soil pH had the greatest positive effect on CUE_ST_, while soil MAT and MAP continued to enhance *R*_h_ (figs. S13 to S15). This divergence in environmental drivers likely underpins the decoupling of CUE_ST_ and *R*_h_ in tropical and temperate regions (fig. S16).

## DISCUSSION

As expected from traditional viewpoints, we found a negative relationship between CUE and *R*_h_, a pattern that primarily occurred in low-productivity arid and cold regions (Fig. 2). This pattern likely arises from a microbial trade-off between C assimilation and respiratory loss under limited plant-derived C inputs. In an environment with limited bioavailable C, microorganisms prioritize C allocation to anabolic processes over respiration (*20*, *26*), resulting in higher CUE relative to *R*_h_. In addition, low temperatures and/or limited water availability in less productive ecosystems (fig. S11) suppress microbial metabolic rates, leading to a low *R*_h_ (*27*). These environmental constraints on water, temperature, and C tightly couple CUE with *R*_h_ in low-productivity ecosystems, likely contributing to the observed negative relationship.

Conversely, in high-productivity ecosystems, the decoupling between CUE and *R*_h_ (Fig. 3) supported our hypothesis that CUE does not continuously decline with increasing *R*_h_ under relatively high C inputs with low nutrient availability (i.e., generally high C:N:P ratios in substrates) (Fig. 1). High-productivity ecosystems, particularly in tropical regions, often experience strong P limitation (*28*–*30*) because of intense weathering, nutrient leaching, and high biotic demand (*31*, *32*). Despite these limitations, microbial communities appear capable of maintaining stoichiometric homeostasis for survival and growth. Long-term environmental filtering and adaptation may allow microorganisms to physiologically acclimate to nutrient scarcity (*33*–*35*). Two key mechanisms likely underpin the observed stability of CUE in high-production environments. First, microorganisms invest C and energy producing extracellular enzymes to acquire limiting nutrients (*24*), a process especially prevalent in tropical soils with high phosphatase activity (*29*, *36*, *37*). Second, microorganisms can exhibit high efficiency in recycling nutrients relative to C (*14*), preserving nutrients for growth while incurring losses of C and energy. These mechanisms help stabilize CUE at a relatively low level, even as *R*_h_ increases under sufficient temperature, water, and C conditions.

This shift in the CUE-*R*_h_ relationship along a productivity gradient carries important implications for soil C retention. In low-productivity ecosystems, high CUE coupled with low *R*_h_ (Fig. 2) suggests a strategy of maximizing C assimilation under limited C inputs, thereby enhancing microbial retention of plant-derived C despite low primary productivity. This finding aligns with decomposition model simulations indicating greater plant-to-soil C transfer efficiency in less productive ecosystems (*38*). In contrast, in high-productivity ecosystems, the decoupling of CUE from *R*_h_ (Fig. 2) implies that microorganisms invest excess C to acquire limiting nutrients, consistent with the frameworks of microbial stoichiometric homeostasis and nutrient mineralization (*39*, *40*). This strategy lowers the retention efficiency of plant-derived C in microbial biomass, leading to higher CO_2_ release through *R*_h_ given high C inputs coupled with limiting nutrient availability.

Our results also suggest divergent trajectories of microbial C retention under various environmental changes. In low-productivity ecosystems (e.g., arid and cold regions), increased primary productivity due to CO_2_ fertilization, warming, or enhanced precipitation (*41*–*43*) could disrupt the current CUE-*R*_h_ coupling. Increased plant C inputs may simultaneously raise *R*_h_ and reduce CUE, amplifying soil C efflux. Thus, vegetation greening in these regions (*41*, *44*) could paradoxically accelerate soil C losses. In contrast, high-productivity ecosystems (e.g., tropical and temperate regions) may show more stable soil C retention because of decoupling between CUE and *R*_h_, regardless of changes in productivity. However, exogenous nutrient inputs such as atmospheric N and P deposition may alter this pattern. The historically low and stable CUE in high-productivity ecosystems could increase if nutrient limitations are alleviated, independent of changes in *R*_h_ (*45*, *46*). Rising atmospheric N and P deposition is enhancing soil nutrient availability, particularly in low-latitude regions (*47*, *48*), potentially boosting soil C sequestration in tropical and temperate ecosystems.

While our findings advance the understanding of microbial controls on soil C cycling, several uncertainties remain. First, we used *R*_h_ observations on the basis of geographic coordinates corresponding to our estimated CUE_ST_ to explore their relationships without accounting for fine-scale spatial and temporal mismatches. Such mismatches could obscure the relationships between microbial C use and respiration, although our sensitivity test still supports the results (figs. S2 and S3). We also acknowledge a potential mismatch in units between the parameters of CUE_ST_ (mass-based) and *R*_h_ (area-based), which could affect the relationship between CUE_ST_ and *R*_h_. However, when *R*_h_ was converted from g C m^−2^ year^−1^ to g C kg^−1^ year^−1^ using soil bulk density, the observed patterns remained consistent (figs. S5 and S6 and table S5). Second, the uneven distribution of paired observations in our study may have also biased the relationships between CUE_ST_ and *R*_h_. CUE_ST_ observations were mainly from China, Europe, and the Americas, with fewer observations from Africa and Russia (Fig. 2A). Further expanding the dataset would therefore be valuable for enhancing the robustness and applicability of the findings. Third, the use of soil pH, cation-exchange capacity (CEC), and clay content as proxies for nutrient availability introduces uncertainty. For example, soil pH differentially influences N and P availability (*49*) and can also affect microbial physiology (*50*–*52*). Last, although N and P are expected to be primarily limiting in temperate and tropical ecosystems, respectively (*28*–*30*), our analysis did not distinguish between their roles. Future research should aim at simultaneously and independently measuring microbial growth and *R*_h_ across temporal and spatial gradients, incorporating direct measures of particular nutrient status. Manipulative isotope-based experiments may also help clarify the trade-off between microbial C assimilation and efflux under resource constraints.

In summary, our study reveals unexpected and contrasting relationships between CUE and *R*_h_ across productivity gradients, supported by multiple lines of evidence, including independent datasets of average daily *R*_h_ and ^13^C/^18^O-derived CUE. In low-productivity ecosystems, the coupling between CUE and *R*_h_ reflects a microbial strategy of prioritizing C assimilation. In contrast, the decoupling observed in high-productivity ecosystems indicates a shift toward nutrient acquisition and the maintenance of stoichiometric homeostasis. These findings imply a limited potential for natural ecosystems to serve as effective soil C sinks under global change (e.g., vegetation greening) and underscore the importance of incorporating microbial metabolic adaptability into future mechanistic assessments of soil C dynamics.

## METHODS

### Data collection

#### Global data of model parameters for estimating CUE_ST_

We compiled a global database of ecoenzymatic activities, microbial biomass, and soil nutrient concentrations in surface soils (mean depth of 11 cm) from a survey of the literature using the Web of Science (http://isiknowledge.com) and the Google Scholar Resource Integrated Database (https://scholar.google.com). Combinations of keywords including “extracellular enzyme,” “exoenzyme,” “ecoenzyme,” “threshold element ratio,” “microbial C use efficiency,” and “enzyme stoichiometry models” were used to search studies published from 1980 to 2022. The criteria for inclusion were as follows: (i) The studies included the activities of C-, N-, and P-acquiring enzymes [β-1, 4-glucosidase (BG), β-1, 4-*N*-acetylglucosaminidase (NAG), l-leucine aminopeptidase (LAP), and acid or alkaline phosphatase (AP)] (table S1); the concentrations of microbial biomass C, N, and P; and the concentrations of soil C [soil organic C (SOC)], N [total N (TN)], and P [total P (TP)], because these indicators are necessary parameters in the stoichiometric model of CUE_ST_ (*24*); (ii) the activities of extracellular enzymes were measured fluorometrically using a 200 μM solution of substrates labeled with 4-methylumbelliferone or 7-amino-4-methylcoumarin; (iii) microbial biomass was determined using chloroform fumigation-extraction; and (iv) data from intensively managed ecosystems (e.g., agroforests, fertilized plantations, sown pastures, croplands, and urban forests) were excluded to avoid influences from anthropogenic disturbances.

On the basis of these criteria, we selected 1094 paired observations across global terrestrial soils at 477 geographic locations from 160 articles (Fig. 2A). The data were extracted from tables or figures of the selected studies using GetData Graph Digitizer software version 2.25. We also recorded corresponding information on site location (longitude and latitude) from the literature. A PRISMA (Preferred Reporting Items for Systematic Reviews and Meta-Analyses) flow diagram (fig. S1) shows the procedure we used for selecting the studies. The dataset was also used by Cui *et al.* (*53*); the present study extends it by pairing each CUE_ST_ with *R*_h_ and conducts new analyses.

#### Global dataset of R_h_

We extracted *R*_h_ values from the Soil Respiration Database (version 5.0; https://daac.ornl.gov/cgi-bin/dsviewer.pl?ds_id=1827) contributed by Jian *et al.* (*25*). A total of 1094 predictions of *R*_h_ were exactly matched to the sampling site coordinates of CUE_ST_ via raster data of *R*_h_. In spite of exact matches in spatial coordinates of sampling sites between CUE_ST_ and *R*_h_, we did not consider the differences in sampling time. However, given that all the sampling sites were from natural ecosystems without anthropogenic nutrient inputs or other major disturbance, the CUE_ST_ and *R*_h_ represent long-term adaptions of microbial communities to the environments in specific ecosystems. These facts should substantially reduce uncertainties in our results caused by mismatches in sampling or measurement times. We also considered that differences in the units of *R*_h_ may alter the relationship between CUE_ST_ and *R*_h_, so we converted the units from g C m^−2^ year^−1^ to g C kg^−1^ year^−1^ via soil bulk density

$$R_{h}\;\left(g\;C\;{\text{kg}}^{-1}\;{\text{year}}^{-1}\right)=R_{h}\;\left(g\;C\;m^{-2}\;{\text{year}}^{-1}\right)/\left(\text{BD}\times h\right)$$

where BD is the soil bulk density (table S10; g cm^−3^), and *h* is the soil depth (m). We defaulted *h* to 0.1 m because the mean soil depth was 0.114 m for the 1094 observations (*53*).

We found that values of mass-based *R*_h_ (g C kg^−1^ year^−1^) were highly correlated with values of area-based *R*_h_ (g C m^−2^ year^−1^) (*P* < 0.001, *R*^2^ = 0.89; fig. S5), generating consistent patterns between CUE_ST_ and *R*_h_ under both *R*_h_ units (Fig. 3 and fig. S6). To minimize the errors caused by uncertainties in soil bulk density and sampling depth during unit conversions, we used the original unit of *R*_h_ (g C m^−2^ year^−1^) instead of converting it to g C kg^−1^ year^−1^ in subsequent analysis.

In addition, we collected 52 observations of average daily *R*_h_ from the 160 selected articles (fig. S7). This means that these studies included all parameters for estimating CUE_ST_ and measured average daily *R*_h_ simultaneously, thus ensuring an exact match between observations of average daily *R*_h_ and CUE_ST_ on both temporal and spatial scales.

#### Environmental variables

To identify the environmental drivers underlying the relationships between CUE_ST_ and *R*_h_, we examined nine variables representing the four basic factors essential to microbial metabolism: temperature, water, C, and nutrients (*27*, *54*, *55*). Specifically, we used mean annual air temperature (MAT) and soil MAT for temperature; MAP for water; LAI, aboveground biomass (AGB), and belowground biomass (BGB) for C; and soil pH, CEC, and clay content as proxies for nutrient availability. In particular, we chose these nutrient-related variables instead of direct soil N and P indicators to avoid collinearity, as soil C, N, and P concentrations are already embedded in the stoichiometric model used to estimate CUE_ST_ (see the “Theoretical basis of the stoichiometric model” section). We retrieved MAT, soil MAT, MAP, LAI, AGB, BGB, CEC, and clay content from multiple sources at a relatively fine spatial resolution (see table S10 for details). In particular, soil pH observations were compiled primarily from the 160 screened studies, which almost always reported soil pH. Only 16 studies did not include soil pH, and we extracted these missing values (*n* = 82) from other recently published studies with the same sample site information and similar geographic coordinates.

Among the nine variables, we further chose four of them (soil temperature, MAP, LAI, and soil pH) as the key indicators representing temperature, water, C, and nutrients, respectively, to explore the specific effects of the four kinds of basic controls on CUE_ST_ and *R*_h_. We choose soil MAT rather than MAT because soil temperatures are generally less variable than atmospheric temperatures (*56*). We used LAI as the key index of C availability for two reasons. First, almost all C sources in surface soil originally come from plant production, especially plant litter inputs. Second, plant litter rather than soil organic matter that has been processed by microorganisms is the dominant C source for microbial acquisition (*57*). Among three proxies of nutrient availability (soil pH, CEC, and clay content), soil pH is a basic regulator of the availabilities of N and P and indirectly represents their supplies (*58*, *59*). We thus adopted it as an indicator of the availabilities of soil N and P.

### Estimating CUE_ST_ using the stoichiometric model

#### Theoretical basis of the stoichiometric model

Sinsabaugh and Follstad Shah (*24*) proposed a biogeochemical-equilibrium model that incorporated ecoenzymatic activities, microbial biomass, and soil resources to estimate CUE_ST_ at the community level. The basis of this stoichiometric approach is using selected ecoenzymatic activities to represent the requirements of microbial resources. Specifically, soil microorganisms synthesize and excrete a series of ecoenzymes (table S1) that degrade organic macromolecules into available substrates (e.g., oligo- and monomers) for microbial assimilation. The profile of ecoenzymatic activity therefore represents the relative microbial acquisition of C, N, and P resources from polymers that balance microbial stoichiometry, given the efficiencies of assimilating elements and resource availability (*24*). Many ecoenzymes contribute to the catabolism of complex polymers (e.g., cellulose), but only a few (i.e., BG, NAG and/or LAP, and AP) catalyze the terminal reactions of the most common substrates and produce soluble products for microbial assimilation. These ecoenzymes thus define the functional interface between product release and acquisition (*60*). They usually have the highest activities per unit microbial biomass and are strongly associated with litter decay and microbial metabolism (*61*). As a result, they are commonly selected as the proximate agents for the acquisition of microbial nutrients during metabolism (*13*, *24*).

#### Estimating CUE_ST_

In detail, we estimated CUE_ST_ using the following equations (*24*)

$${\text{CUE}}_{\text{ST}}={\text{CUE}}^{\text{max}}\times {\left\{\frac{\left(S_{C:N}\times S_{C:P}\right)}{\left[\left(K_{C:N}+S_{C:N}\right)\times \left(K_{C:P}+S_{C:P}\right)\right]}\right\}}^{0.5}$$ (1)

$$S_{C:N}=\frac{B_{C:N}}{L_{C:N}}\times \frac{1}{{\text{EEA}}_{C:N}}$$ (2)

$$S_{C:P}=\frac{B_{C:P}}{L_{C:P}}\times \frac{1}{{\text{EEA}}_{C:P}}$$ (3)

where $S_{C:N}$ and $S_{C:P}$ are scalars that represent the extent to which the allocation of extracellular enzyme activity (EEA) offsets the disparity between the elemental composition of available resources and the composition of microbial biomass. In this case, ${\text{EEA}}_{C:N}$ and ${\text{EEA}}_{C:P}$ were calculated as BG/(NAG + LAP) and BG/AP, respectively. Furthermore, *L*_C:N_ and *L*_C:P_ were estimated as molar ratios of SOC:TN and SOC:TP, respectively, and *B*_C:N_ and *B*_C:P_ were calculated as molar ratios of MBC (microbial biomass C):MBN (microbial biomass N) and MBC:MBP (microbial biomass P), respectively. Both $K_{C:N}$ and $K_{C:P}$ are half-saturation constants for CUE_ST_ based on the availabilities of C, N, and P, assumed to be 0.5 (*24*). ${\text{CUE}}^{\text{max}}$ (maximum CUE) is about 0.6 based on metabolic kinetics and energetics (*19*, *39*).

It is worth noting that the enzymatic activities we selected were generally proxies of microbial metabolism using polymeric organic matter (*24*, *61*). Soluble resources not requiring enzymatic catalysis for acquisition could potentially skew our estimates (*3*, *62*). We thus only retained observations of surface soils from natural ecosystems, where the original dominant nutrient pools should be polymer-rich organic matter from plant litter (*63*). This filtering could minimize uncertainties introduced by soluble resources such as rhizodeposition and fertilizers.

### Dataset of ^13^C/^18^O-based CUE for global natural ecosystems

For verifying the results relevant to CUE_ST_, we also considered the results of CUE independently measured by the isotope-based approaches. We obtained a dataset of ^13^C/^18^O-CUE at a global scale (fig. S7) from a recent study based on the meta-analysis (*6*). This study collected 132 observations of ^13^C/^18^O-CUE measured at 46 locations from 16 publications. In cases of manipulation experiments (for example, fertilization experiments), only data from control plots was included. Therefore, the dataset only represents an investigation of CUE in natural ecosystems.

### Statistical analyses

The locations of these sample sites for CUE_ST_, *R*_h_, and ^13^C/^18^O-CUE were divided into four climatic zones (tropical, temperate, arid, and cold zones) on the basis of the global Köppen-Geiger grid map of climatic classification (*64*). We identified the relationships between CUE_ST_ (or ^13^C/^18^O-CUE) and *R*_h_ (or average daily *R*_h_) among climate zones and between low-productivity (LAI < 3) and high-productivity (LAI > 3) ecosystems using generalized linear models (Figs. 2 to 4). We also determined the patterns of decoupling between CUE_ST_ and *R*_h_ (or average daily *R*_h_) at the global scale by first identifying their decoupling thresholds from a piecewise linear-regression analysis (Figs. 2C and 3C, fig. S6, and tables S4 to S6). The regression relationships between CUE_ST_ and *R*_h_ (or average daily *R*_h_) were fitted with linear models using the “segmented” (version 2.1-3) R package (*65*). The confidence intervals of the thresholds were calculated using 1000 bootstrap samples and the “SiZer” (version 0.1-8) R package (*66*). In addition, we tested three nonlinear functions (exponential, logarithmic, and quadratic) for the relationships of CUE_ST_ versus *R*_h_ (fig. S4) and CUE_ST_ versus average daily *R*_h_ (fig. S8), fitted separately within low- and high-productivity ecosystems. These analyses corroborate the productivity-dependent relationships between CUE_ST_ and *R*_h_: robust negative associations in low-productivity ecosystems and weak or near-zero relationships in high-productivity ecosystems.

To gauge bias from coordinate-based pairing, we ran a stratified random-removal sensitivity test using the “tidyverse” (version 2.0.0) and “broom” (version 1.0.7) R packages: We repeatedly removed 20% of CUE_ST_-*R*_h_ pairs within climatic zones and separately within productivity classes and reidentified slopes of regressions between CUE_ST_ and *R*_h_. The negative relationship persisted in arid and cold zones or in low-productivity ecosystems and remained weak or indistinguishable from zero in temperate and tropical zones or in high-productivity ecosystems (figs. S2 and S3 and tables S2 and S3), indicating that our conclusions are robust to plausible mismatching.

We further investigated the effects of resource availabilities on the relationships between CUE_ST_ and *R*_h_ for low- and high-productivity ecosystems. First, we examined the distribution of the newly defined variable (CUE_ST_/*R*_h_) with productivity groups by kernel density plots and formal normality tests. Both the plots and test results indicated that CUE_ST_/*R*_h_ deviate from normality (Shapiro-Wilk, Lilliefors, and Anderson-Darling, all *P* < 0.001; fig. S9 and table S7). We therefore further verified robustness with (i) ordinary least squares on log-transformed CUE_ST_/*R*_h_ with HC3 robust standard errors and (ii) gamma generalized linear models (log link) using the “lmtest” and “sandwich” (version 3.1-1) R packages (fig. S10 and tables S8 and S9). Second, an analysis of mixed-effects model selection was adopted to identify the most important predictors among the nine environmental variables affecting CUE_ST_/*R*_h_ using the “glmulti” (version 1.0.8) R package (*67*). The model selection was based on maximum-likelihood estimation. The importance of each predictor was calculated as the sum of Akaike weights for models that included this predictor (Fig. 4, A and B, and fig. S12). A cutoff of 0.8 was set to differentiate between essential and nonessential predictors (*67*). The signs and relative importance patterns of nine environmental variables identified by robustness analysis were consistent with the results of mixed-effects models, which suggests that the analysis of mixed-effects model selection is reasonable and robust. Third, we separately identified the relationships between CUE_ST_/*R*_h_ and nine environmental variables for low- and high-productivity ecosystems and for four climate zones using generalized linear models (Fig. 4C and fig. S12).

We also explored possible mechanisms affecting the relationships between CUE_ST_ and *R*_h_ by identifying the effects of the four key variables of the nine environmental variables on CUE_ST_ and *R*_h_ using multiple statistical analyses. First, a linear mixed-effects model was used to analyze the differences of the four variables among climatic zones (fig. S11). The model was constructed using the “lme” function from the “nlme” (version 3.1-164) R package, with “climatic zone” as the fixed factor and “sampling site” as the random factor. Tukey’s tests were further used to identify the significance of differences in variables among the climatic zones using the “multcompView” (version 0.1-10) R package. Second, the analysis of mixed-effects model selection was adopted to identify the most important predictors among the four key variables affecting CUE_ST_/*R*_h_, CUE_ST_, and *R*_h_ separately (figs. S12 and S15). Third, the partial correlation analysis further identified specific relationships between each variable and CUE_ST_ (or *R*_h_) by controlling the other three variables using the “pcor.test” function in the “ppcor” (version 1.1) R package (fig. S13). Fourth, we also used a variation-partitioning analysis to quantify the independent and joint influences of the four key variables on CUE_ST_ with *R*_h_ using the “varpart” function in the “vegan” (version 2.6-8) R package (fig. S12). We calculated the relative independent and joint influences of the four variables on CUE_ST_ and *R*_h_ to facilitate interpretation

$$\text{Independent relative influence}=\frac{\text{Independent explanation of each variable}}{\text{Total explanation of}\;\text{all}\;\text{variables}}$$

$$\text{Joint relative influence}=\frac{\text{Joint explanation of}\;\text{one}\;\text{with others}}{\text{Total explanation of}\;\text{all}\;\text{variables}}$$

All statistical analyses were performed using R software (version 4.4.2 for the original analyses and version 4.5.1 for the revision) (*68*).

## Supplementary Materials

### This PDF file includes:

Figs. S1 to S16 Tables S1 to S10 References
