# Supplementary material for: Productivity-driven decoupling of microbial carbon use efficiency and respiration across global soils
Source: Sci Adv. 2026 Jan 14;12(3):eadz5319. doi: 10.1126/sciadv.adz5319 (PMC12802823; doi:10.1126/sciadv.adz5319)
Supplement: Supplementary file 1 — Figs. S1 to S16 Tables S1 to S10 References [file sciadv.adz5319_sm.pdf]

Science Advances
AAAS

Supplementary Materials for

Productivity-driven decoupling of microbial carbon use efficiency and respiration across global soils

Yongxing Cui *et al.*

Corresponding author: Yongxing Cui, cuiyongxing@zedat.fu-berlin.de; Ji Chen, chenji@ieecas.cn

Sci. Adv. **12**, eadz5319 (2026)
DOI: 10.1126/sciadv.adz5319

This PDF file includes:

Figs. S1 to S16
Tables S1 to S10
References

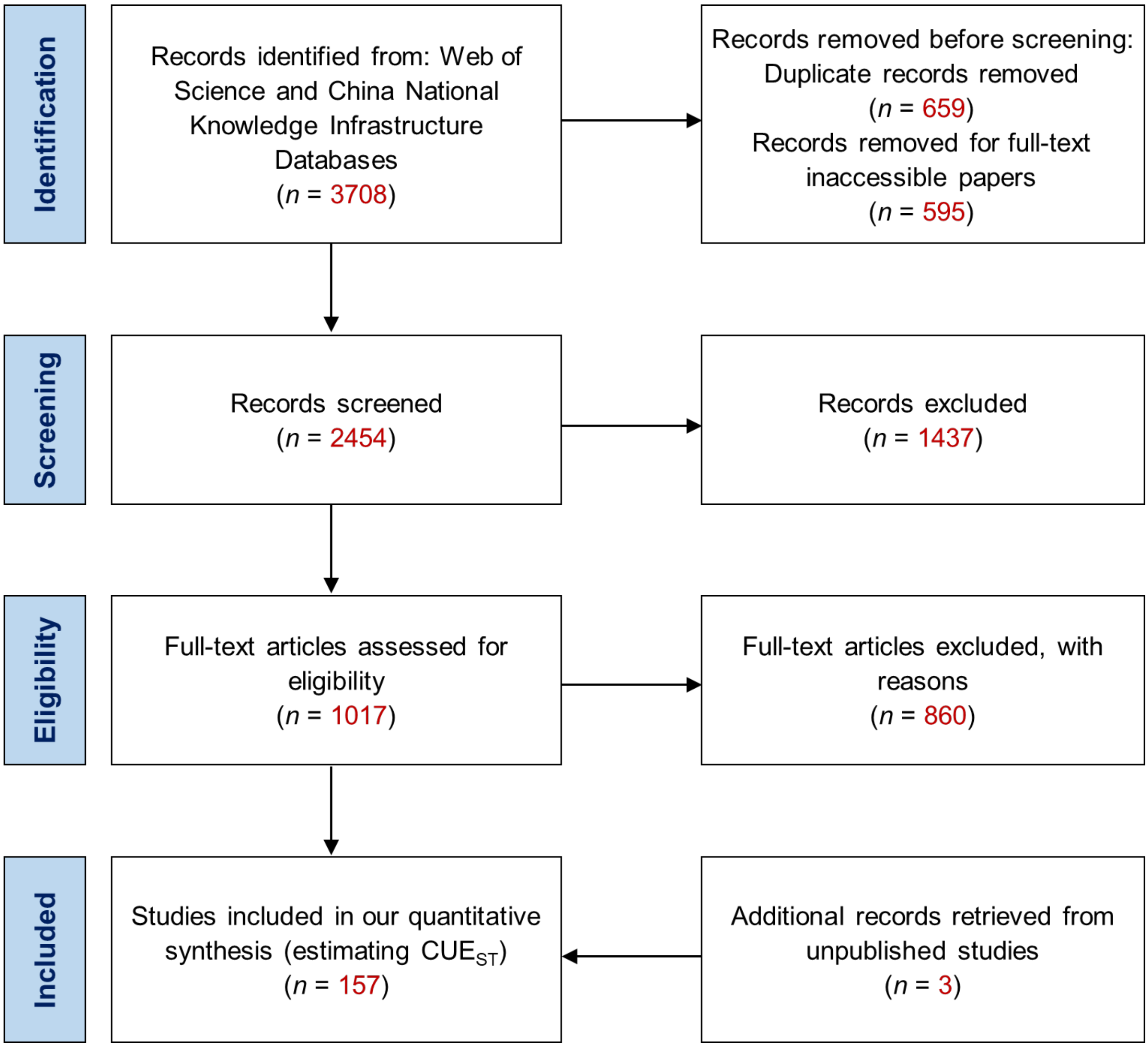

Fig. S1 PRISMA flow diagram of the procedure used for the selection of studies for estimating microbial C use efficiency (CUE_{ST}).

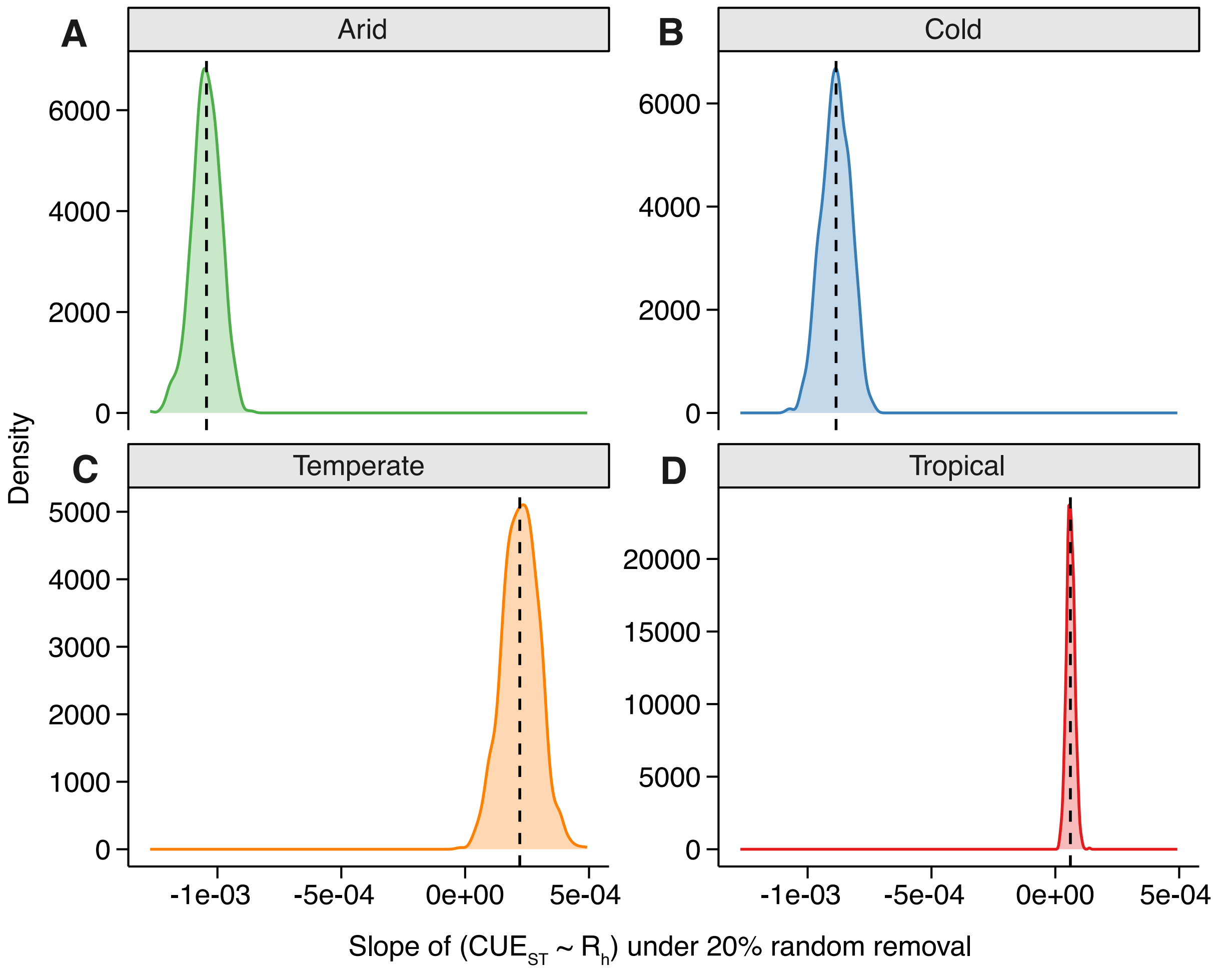

Fig. S2 Robustness of regression slopes of CUE_{ST}-R_h by climatic zone under random 20% removal.

Distributions of bootstrapped slopes ($CUE_{ST} \sim R_h$) for arid, cold, temperate, tropical zones (B = 1000 iterations); dashed vertical lines indicate the baseline slopes from the full dataset. The negative slopes in arid and cold zones are recovered in 100% of iterations and are significant in 100%; slopes in temperate and tropical zones are small and rarely significant, consistent with weak or near-zero relationships.

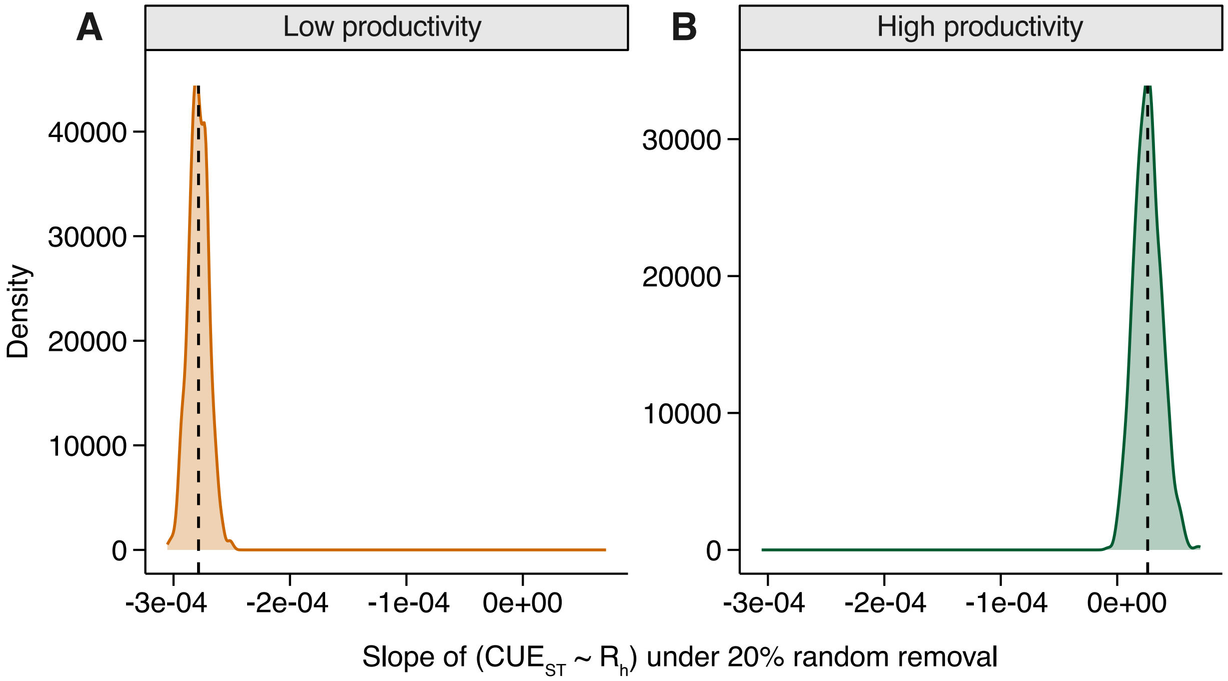

Fig. S3 Robustness of regression slopes of CUE_{ST} ~ R_h by ecosystem productivity under random 20% removal.

Distributions of bootstrapped slopes (CUE_{ST} ~ R_h) for low and high productivity ecosystems (*B* = 1000 iterations); dashed vertical lines indicate the baseline slopes from the full dataset. The negative slope in low-productivity ecosystems is recovered and significant in 100% of iterations; slopes in high-productivity ecosystems remain near zero.

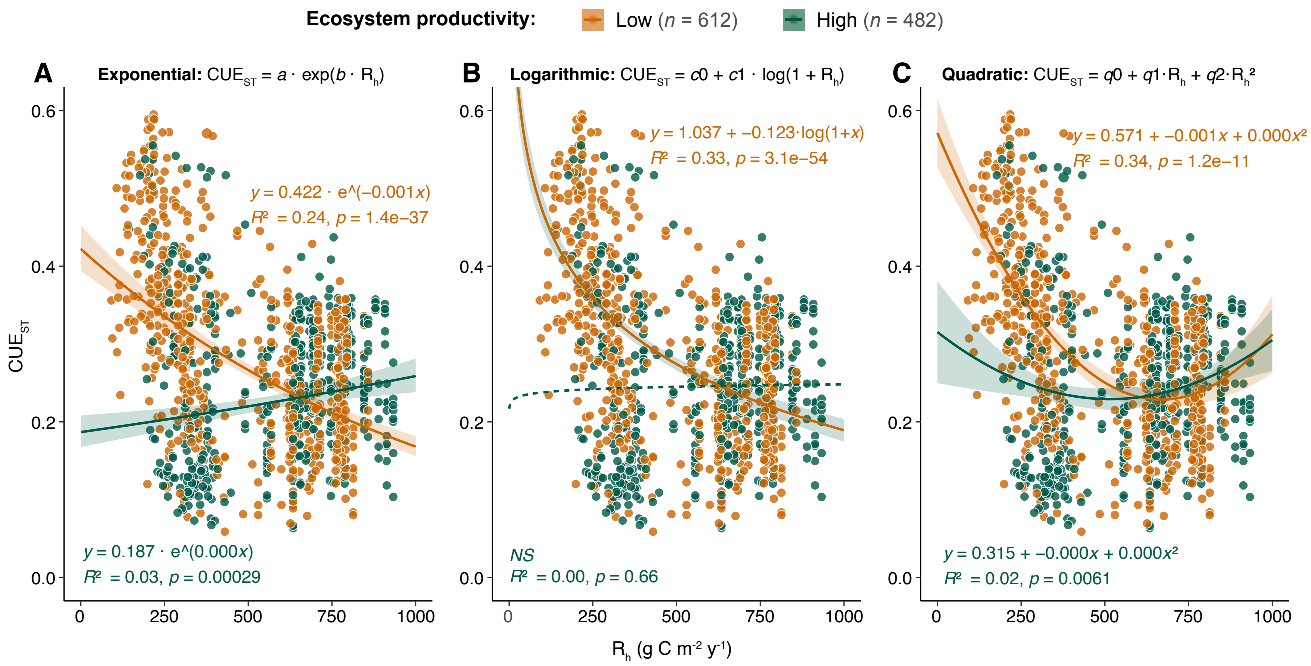

Fig. S4 Nonlinear fits (exponential, logarithmic, and quadratic) of $CUE_{ST} \sim R_h$ by productivity. Scatter points show low- (orange) and high- (green) productivity ecosystems. Curves are the fitted exponential (**A**), logarithmic (**B**), and quadratic (**C**) models, each with 95% confidence interval. Panel annotations report the fitted equation, R^2, and p-value for each productivity class. Across model forms, low-productivity ecosystems exhibit higher R^2 and significant relationships between them, whereas high-productivity ecosystems remain weak (low R^2) or non-significant relationships.

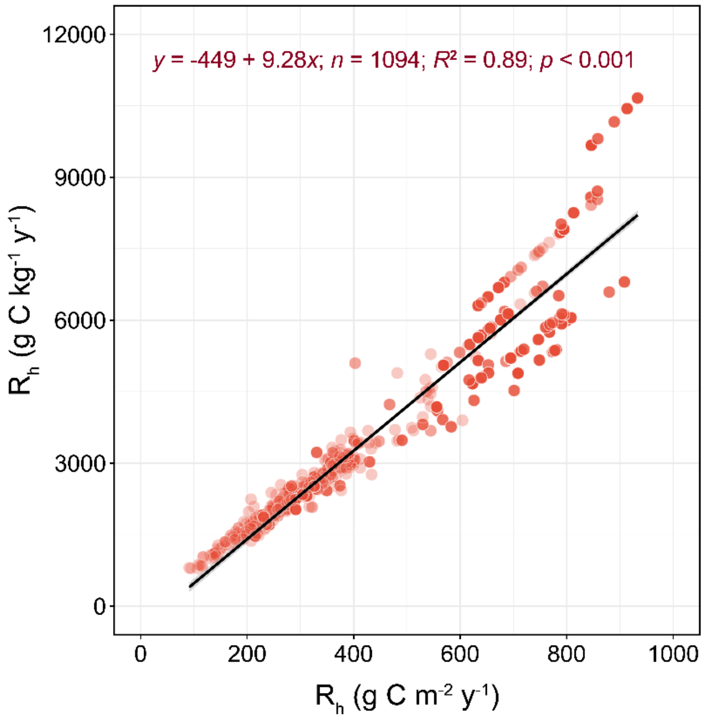

Fig. S5 Consistency of annual heterotrophic respiration rate (R_h) of global soils using two units (g C m^{-2} y^{-1} and g C kg^{-1} soil y^{-1}) for 1094 observations at 447 sites.

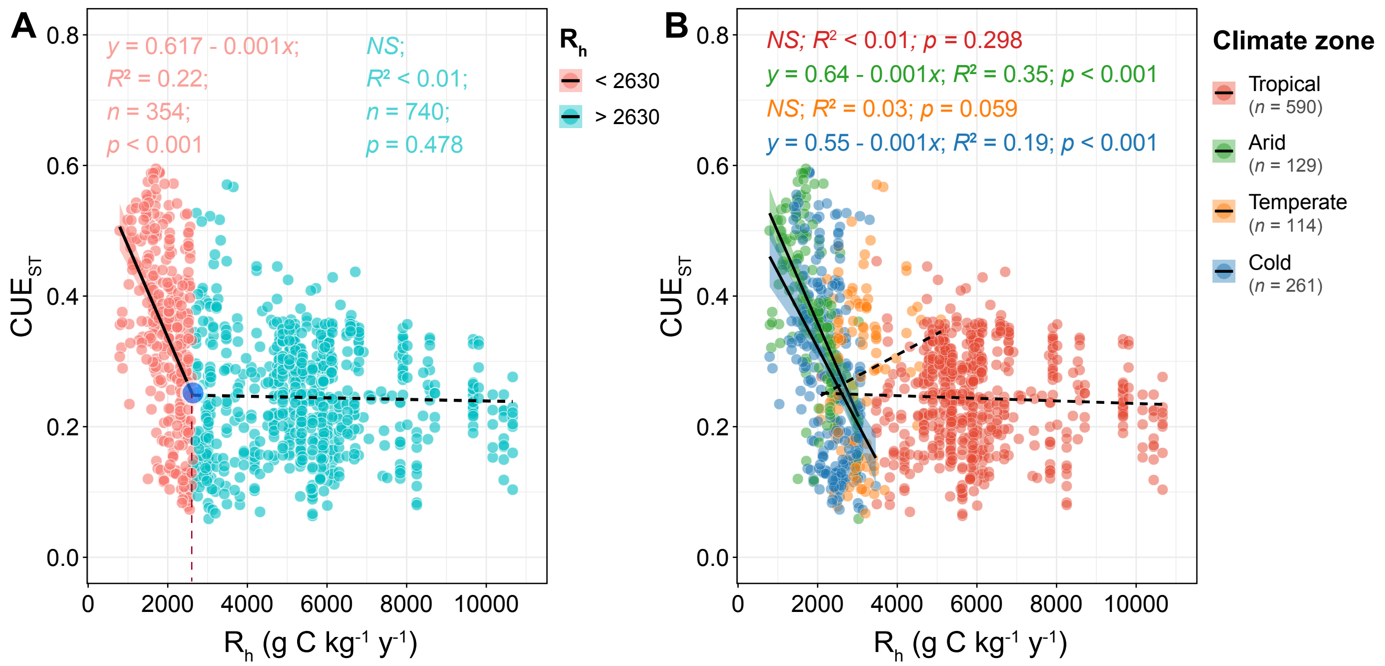

Fig. S6 Relationships between microbial C use efficiency (CUE_{ST}) and average annual heterotrophic respiration rate (R_h, g C kg^{-1} soil y^{-1}) for global soils, in the case of changing the unit of R_h.

(**A**) Decoupling of CUE_{ST} with R_h occurs as R_h increased beyond 2630 g C kg^{-1} soil y^{-1}. The R_h breakpoint (2630 g C kg^{-1} soil y^{-1}) was estimated using piecewise regression analyses, and relationships of CUE_{ST} with R_h before and after the R_h breakpoint were identified using generalized linear models (adjusted-R^2 = 0.25, n = 1094, and $p < 0.001$ in the piecewise regression analyses). The shaded circle indicates breakpoint R_h, and the shaded areas are the 97.5% confidence intervals of the breakpoint. Solid black lines indicate model fits between CUE_{ST} and R_h ($p < 0.05$). Details in the execution and results of the piecewise regression analyses can be seen in the Supplemental Table S5. (**B**) Relationships between CUE_{ST} and R_h at different climate zones were identified using generalized linear models at $p < 0.05$. The shaded area is the 97.5% confidence interval of the linear regressions. All continuous lines are significant at $p < 0.05$, whereas dashed lines are not significant ($p > 0.05$).

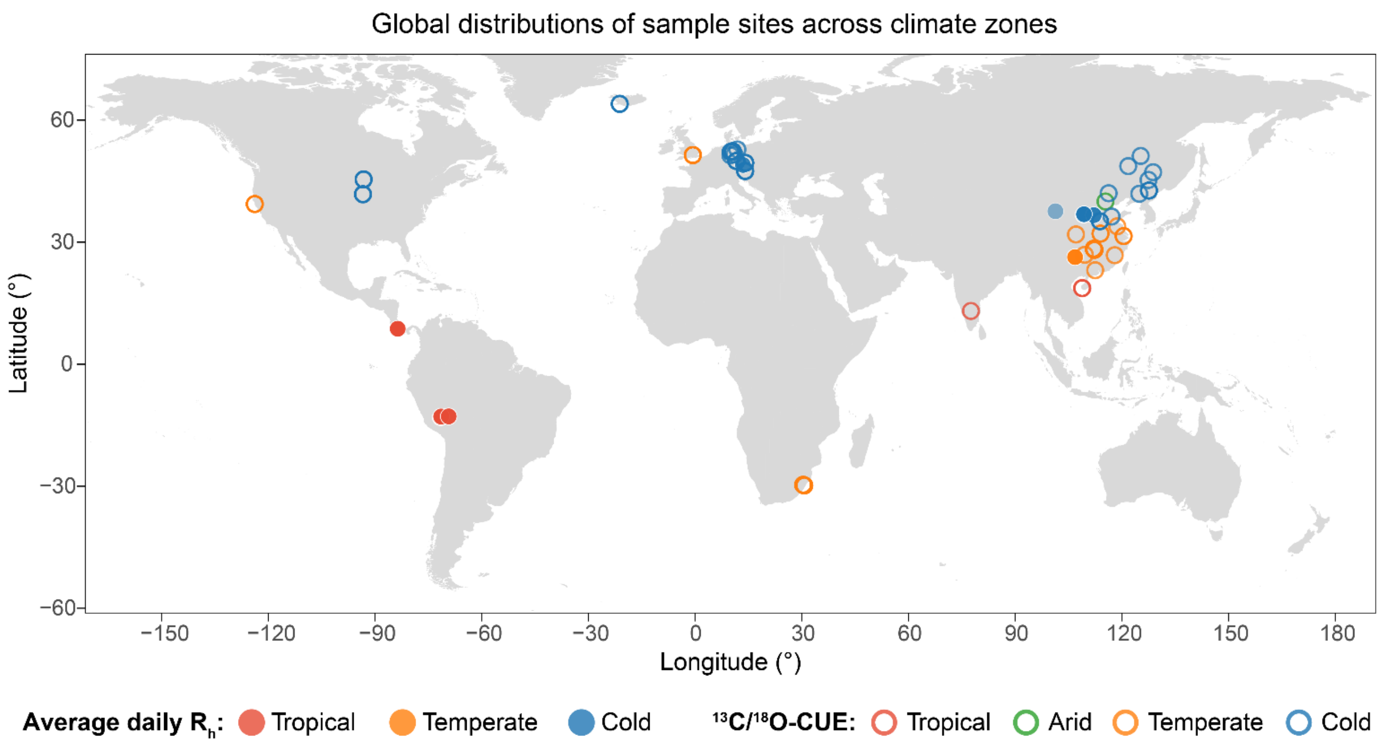

Fig. S7 Global geographical distribution of sample sites for average daily heterotrophic respiration rate (average daily R_h, n = 52) and $^{13}C/^{18}O$-CUE (n = 132).

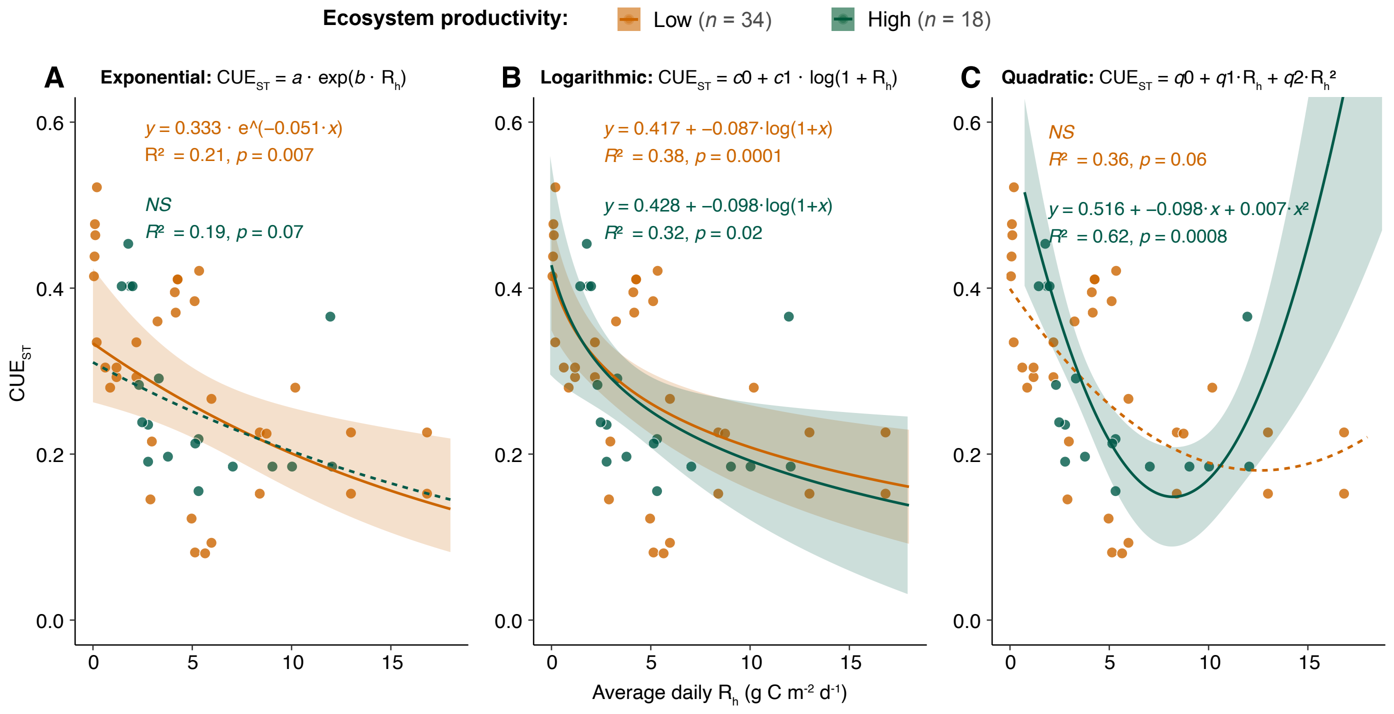

Fig. S8 Nonlinear fits (exponential, logarithmic, and quadratic) of CUE_{ST} ~ average daily R_h by productivity.

Scatter points show low- (orange) and high- (green) productivity ecosystems. Curves are the fitted exponential (**A**), logarithmic (**B**), and quadratic (**C**) models, each with 95% confidence interval. Panel annotations report the fitted equation, R^2, and p-value for each productivity class. Across model forms, low-productivity ecosystems exhibit higher R^2 and significant relationships between them, whereas high-productivity ecosystems remain weak (low R^2) or non-significant relationships.

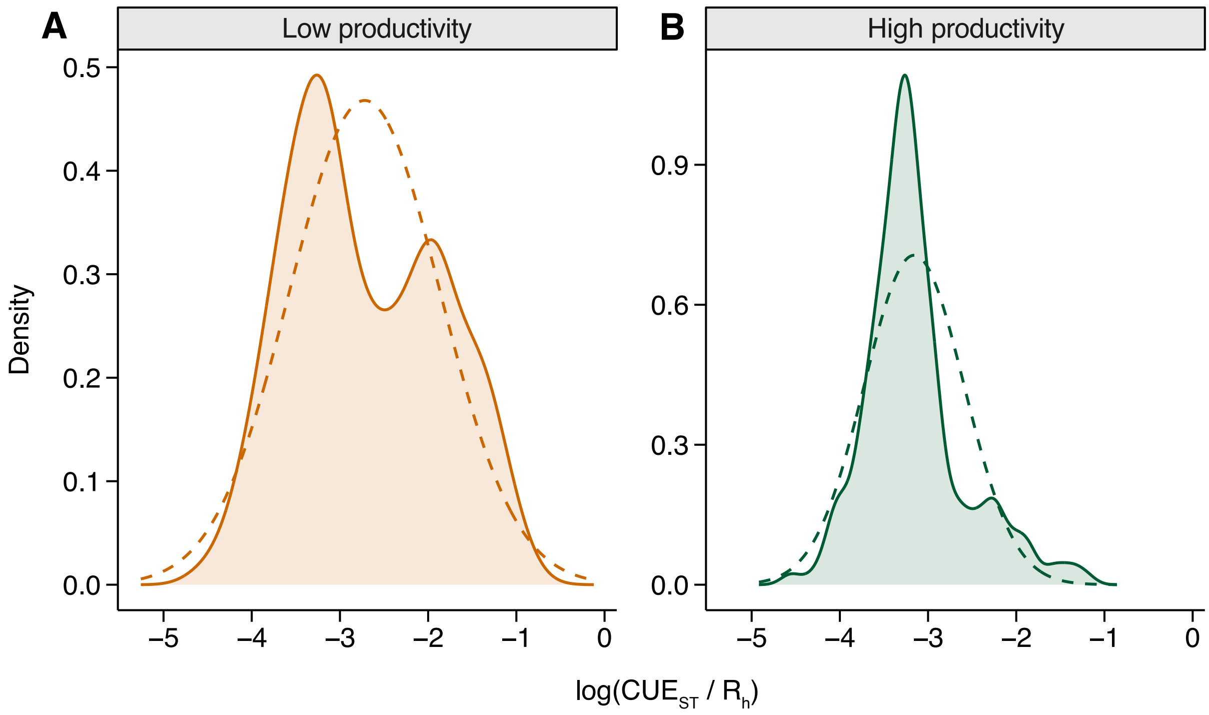

Fig. S9 Kernel density distribution of log-transformed CUE_{ST}/R_h by productivity (low *vs* high productivity ecosystems), with fitted normal curves (dashed).

Both groups deviated from normality (see Table S7), motivating robust modeling choices.

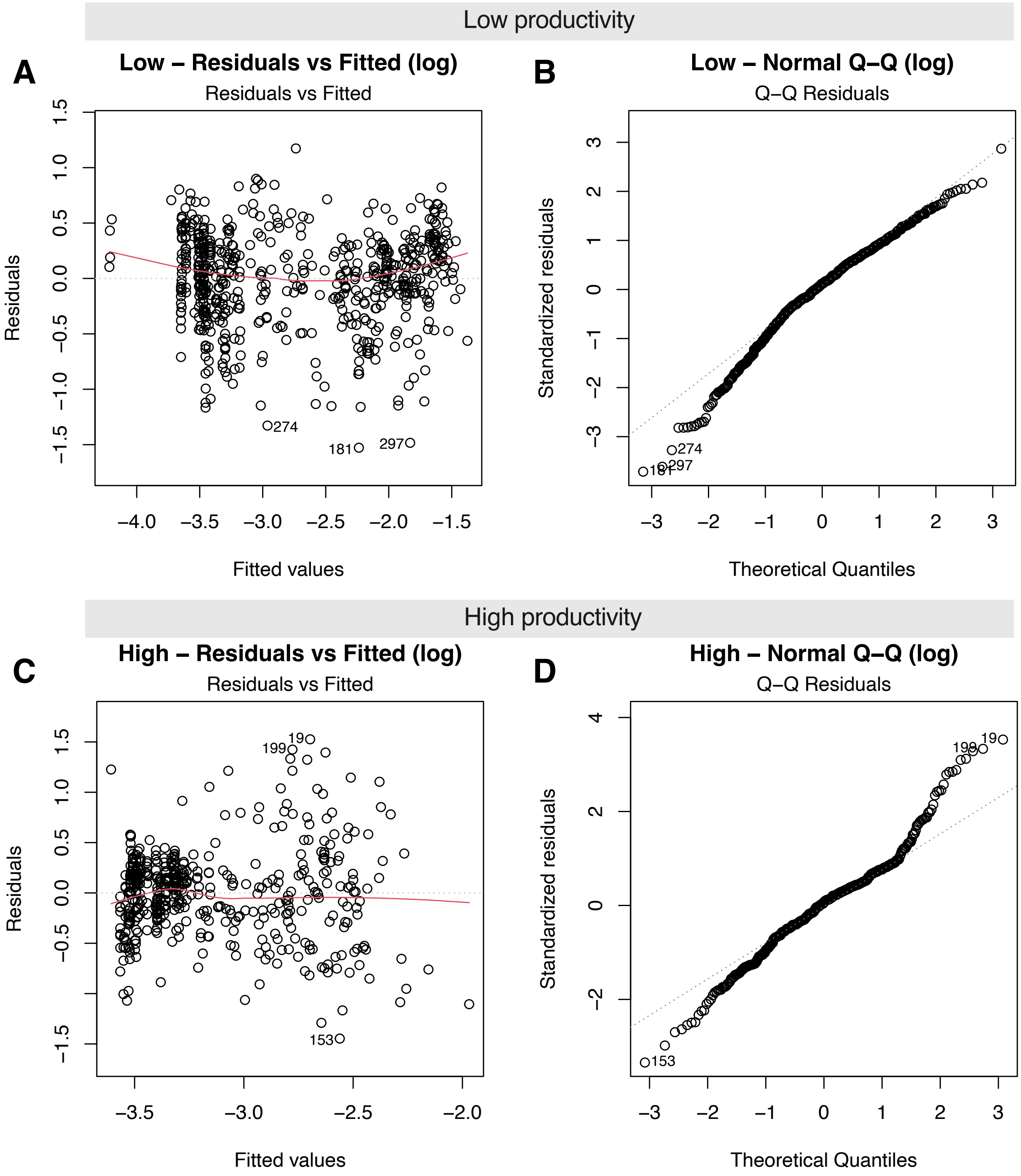

Fig. S10 Residual diagnostics for Gaussian linear models on log-transformed CUE_{ST}/R_h by productivity (low *vs* high productivity ecosystems).

Left: residuals *vs* fitted; right: Normal Q-Q plot. Patterns indicate deviations from normality and heteroscedasticity, for which HC3-robust errors and Gamma models are used as robustness checks.

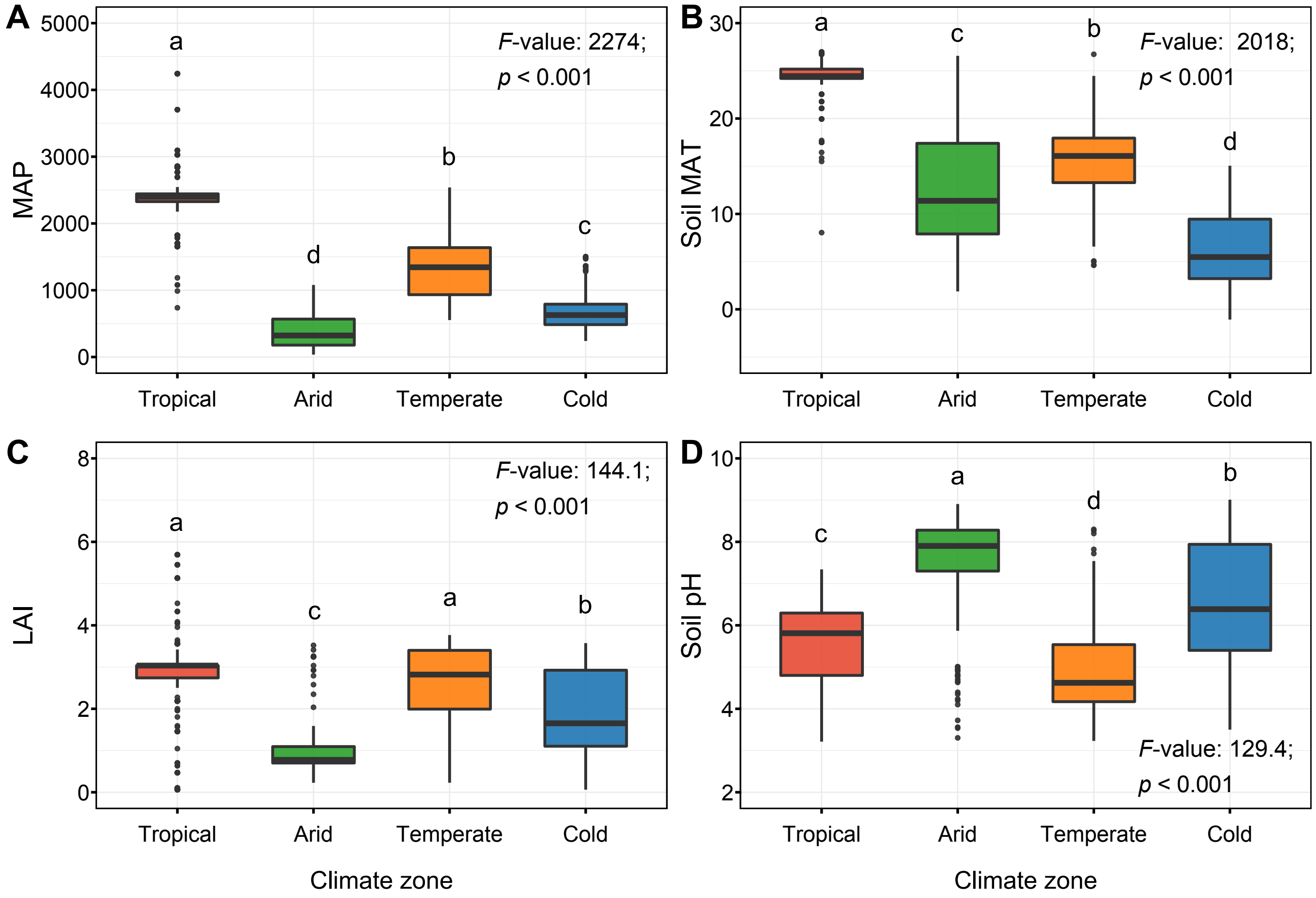

Fig. S11 Boxplots the differences of the four critical environmental variables among the four climate zones for 1094 observations at the global scale.

Different letters indicate significant differences ($p < 0.001$) among the climate zones based on a linear mixed-effects model followed by a Tukey's test. This result is also shown in our previous study (*53*).

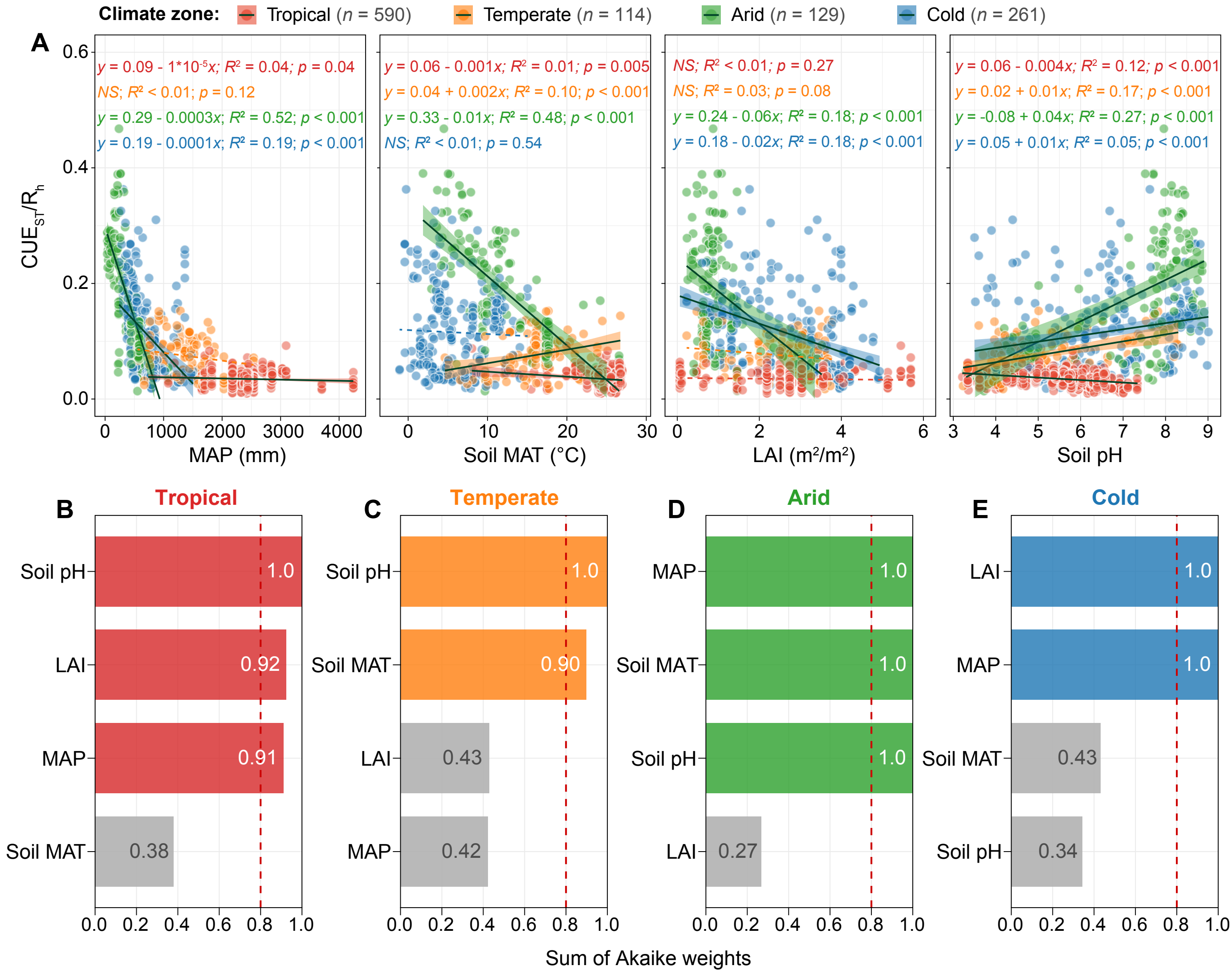

Fig. S12 Effects of four key environmental variables on the relative change of microbial C use efficiency (CUE_{ST}) *vs* average annual heterotrophic respiration rate (R_h) (CUE_{ST}/R_h) among climate zones.

(**A**) The effects of these environmental variables on CUE_{ST}/R_h among climate zones were also evaluated using generalized linear models. The shaded area is the 97.5% confidence interval of the linear regressions. All continuous lines are significant at $p < 0.05$, whereas dashed lines are not significant ($p > 0.05$). (**B-E**) The analysis of mixed-effects model selection was used to evaluate the relative importance of these variables affecting CUE_{ST}/R_h. Values of the sum of Akaike weights were estimated based on corrected Akaike's information criteria. A threshold value of 0.8 (red dashed line) was set to identify the most important variables. The relative change of CUE_{ST} *vs* R_h is expressed by CUE_{ST}:R_h ratio (CUE_{ST}/R_h). mean annual soil temperature, soil MAT; mean annual precipitation, MAP; leaf area index, LAI.

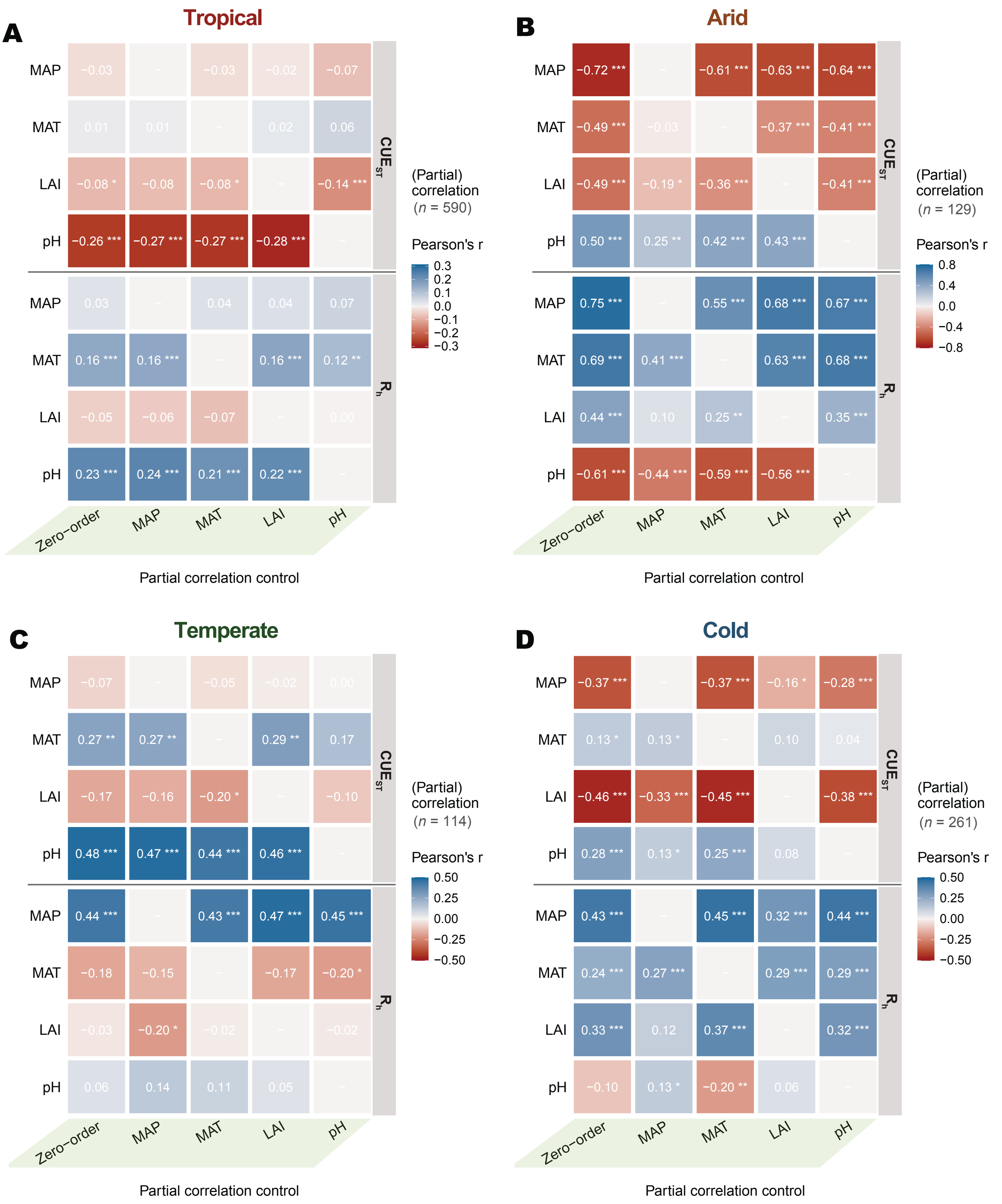

Fig. S13 Partial correlation analysis of microbial C use efficiency (CUE_{ST}) and average annual heterotrophic respiration rate (R_h) with MAP, MAT, LAI, and soil pH, respectively, among four climate zones.

MAP, mean annual precipitation; MAT, soil mean annual temperature; LAI, leaf area index.

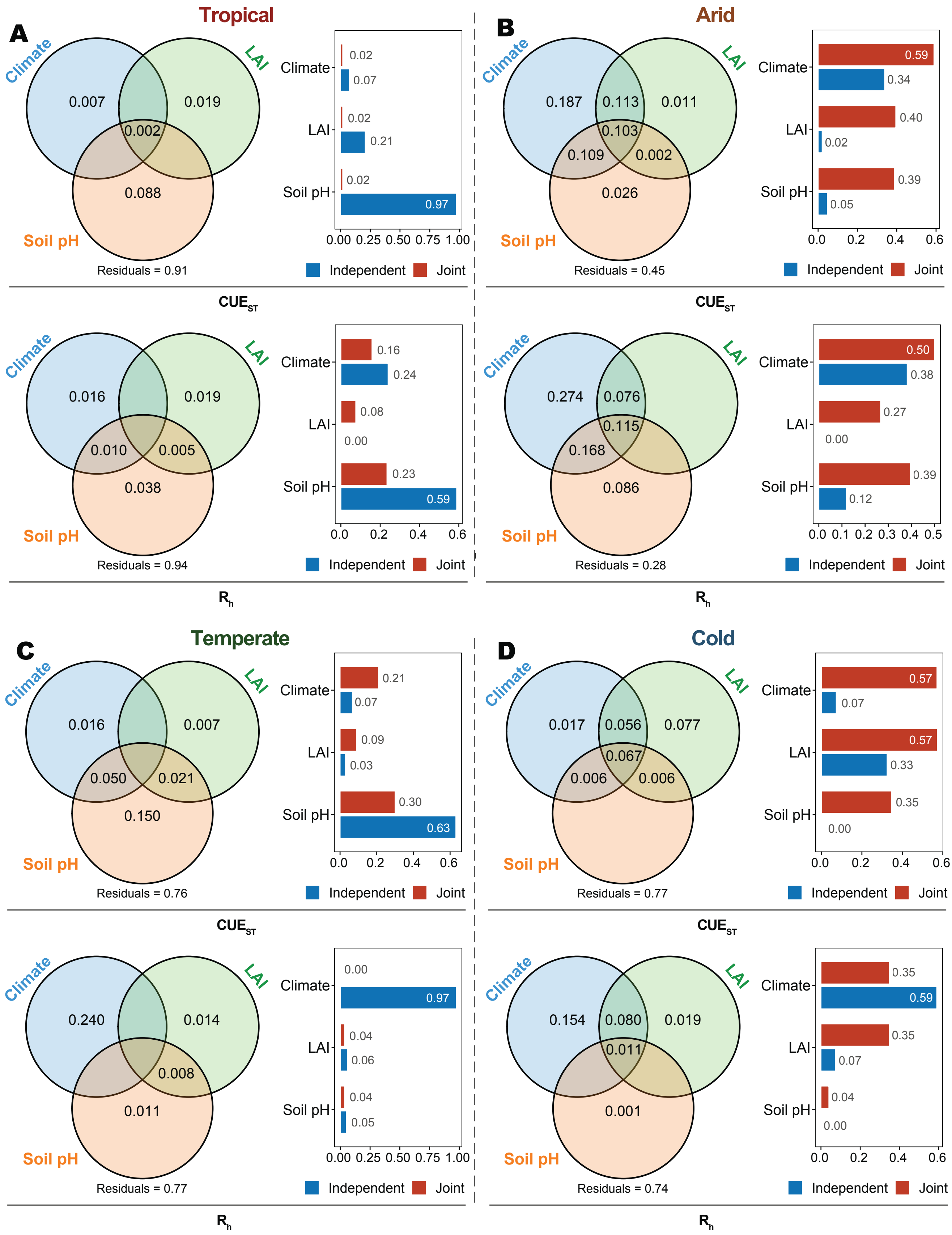

Fig. S14 Variation-partitioning analysis of the effects of climate (MAP and MAT), LAI, and soil pH on microbial C use efficiency (CUE_{ST}) and average annual heterotrophic respiration rate (R_h), respectively, across four climate zones.

The bar charts on the right show independent and joint relative influences for climate (MAP and soil MAT), LAI, and soil pH on CUE_{ST} and R_h. MAP, mean annual precipitation; MAT, soil mean annual temperature; LAI, leaf area index.

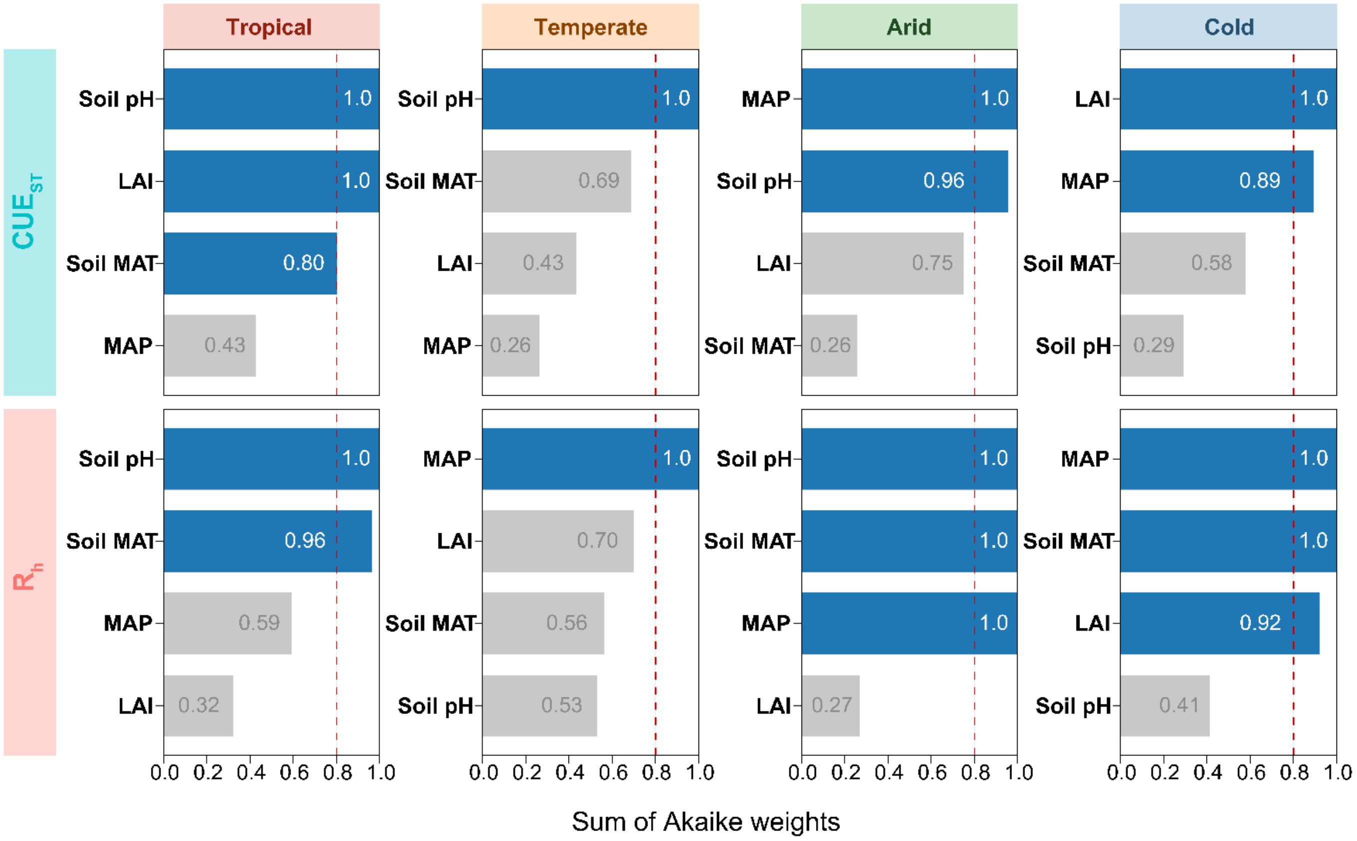

Fig. S15 Effects of four key environmental variables on microbial C use efficiency (CUE_{ST}) and average annual heterotrophic respiration rate (R_h) among climate zones.

The analysis of mixed-effects model selection was used to evaluate the relative importance of these variables affecting CUE_{ST} and R_h, respectively. Values of the sum of Akaike weights were estimated based on corrected Akaike's information criteria. A threshold value of 0.8 (red dashed line) was set to identify the most important variables. mean annual soil temperature, soil MAT; mean annual precipitation, MAP; leaf area index, LAI.

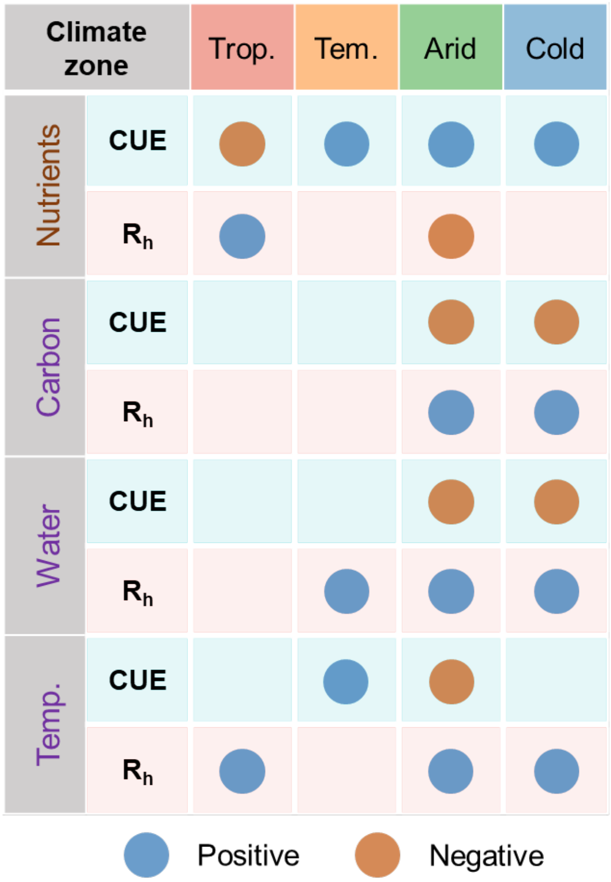

Fig. S16 Conceptual summary based on Supplemental Figs. S12-S15 shows key environmental variables affecting microbial C use efficiency (CUE_{ST}) and annual heterotrophic respiration rate (R_h) across climate zones.

Blue circles indicate positive influences of resource variables on CUE_{ST} or R_h, and yellow circles indicate negative influences of resource variables on CUE_{ST} or R_h. Resource variables include temperature, water, C, and nutrients (mainly N and P). Trop., tropical zone; Tem., temperate zone; Arid, arid zone; Cold, cold zone.

Table S1
Extracellular enzymes commonly used to assess the activities of ecoenzymes involved in the cycling of soil C, nitrogen (N), and phosphorus (P).

Ecoenzymes	Abbrevia	EC[a]	Function
β-1, 4-glucosidase	BG	3.2.1.21	Cellulose degradation: hydrolyzes glucose from cellobiose
β-1, 4-N-acetylglucosaminidase	NAG	3.2.1.14	Chitin and peptidoglycan degradation: hydrolyzes glucosamine from chitobiose
L-leucine aminopeptidase	LAP	3.4.11.1	Proteolysis: hydrolyzes leucine and other hydrophobic amino acids from the N terminus of polypeptides
Acid or alkaline phosphatase	AP	3.1.3.1	Hydrolyzes phosphate from phosphosaccharides and phospholipids

Note: Enzyme commission classification (*60*).

Table S2

Summary of robustness metrics of regression slopes of $CUE_{ST} \sim R_h$ by climatic zone (random 20% removal).

	Baseline slopes by climatic zones			Robustness summary (random 20% removal) by climatic zones					
Climatic zone	base_est	base_*p*	base_*n*	*n*_iter	same_sign_pct	pct_*p*_lt_0_05	median_slope	IQR_slope	median_delta
Arid	-1.04e-03	6.32e-16	129	1000	100	100	-1.04e-03	7.64e-05	-6.65e-07
Cold	-8.85e-04	2.88e-13	261	1000	100	100	-8.85e-04	8.18e-05	-3.11e-07
Temperate	2.21e-04	2.51e-01	114	1000	99.9	0.5	2.24e-04	9.97e-05	3.62e-06
Tropical	6.08e-05	4.91e-02	590	1000	100	34.5	6.12e-05	2.19e-05	4.01e-07

Note: Fields: group, *n*_iter, same_sign_pct, pct_*p*_lt_0_05, median_slope, IQR_slope, median_delta (bootstrapped slope - baseline slope). One-line interpretation: Arid/Cold: 100% sign & 100% significant; Temperate: 99.9% sign, 0.5% significant; Tropical: 100% sign, 34.5% significant; median $\Delta \approx 10^{-6}$ magnitude.

Table S3

Summary of robustness metrics of regression slopes of CUE_{ST} ~ R_h by productivity (random 20% removal).

	Baseline slopes by ecosystem productivity			Robustness summary (random 20% removal) by ecosystem productivity					
Productivity	base_est	base_*p*	base_*n*	*n*_iter	same_sign_pct	pct_*p*_lt_0_05	median_slope	IQR_slope	median_delta
Low	-2.79e-04	5.95e-48	612	1000	100	100	-2.79e-04	1.14e-05	-2.21e-07
High	2.62e-05	2.04e-01	482	1000	99.7	5.8	2.60e-05	1.55e-05	-2.07e-07

Note: Fields: group, *n*_iter, same_sign_pct, pct_*p*_lt_0_05, median_slope, IQR_slope, median_delta (bootstrapped slope - baseline slope). One- line interpretation: Low: 100% sign & 100% significant; High: 99.7% sign, 5.8% significant; median $\Delta \approx 2\times10^{-7}$ magnitude.

Table S4:

Execution and results of the piecewise regression analyses for identifying relationships between microbial C use efficiency (CUE_{ST}) and annual heterotrophic respiration rate (R_h, g C m^{-2} y^{-1}) (corresponding to Fig. 2D).

First step:

Calculate the adjusted R^2 and p of the piecewise linear regression using the "segmented" package

#Fitting a simple linear regression model between CUE_{ST} and R_h first
fit_lm <- lm(CUE_{ST} ~R_h, data = df1)
summary(fit_lm)

#In the above known linear regression model, the possible breakpoints were found by the "segmented" function.
> lm_seg1 <- segmented(fit_lm, seg.Z = ~ R_h, npsi = 1)
> summary(lm_seg1)
Call:
segmented.lm(obj = fit_lm, seg.Z = ~ R_h, npsi = 1)

Estimated breakpoint(s):

	Est.	St.Err
psi1.R_h:	**340.306**	10.835

Meaningful coefficients of the linear terms:

	Estimate	Std. Error	*t*	Pr(>|t|)	Significance
(Intercept)	6.15e-01	2.30e-02	26.73	<2e-16	***
R_h	-1.10e-03	8.80e-05	-12.5	<2e-16	***
U1.R_h	1.11e-03	9.09e-05	12.23	NA	

***, $P < 0.001$
Residual standard error: 0.09684 on 1090 degrees of freedom
Multiple R^2 = 0.2508, adjusted R^2 = **0.2487**

Boot restarting based on six samples. Last fit:
Convergence attained in two iterations (rel. change 5.1627e-16)

Second step:

Calculate the confidence interval of piecewise linear regression using the "SiZer" package

#The breakpoint of the piecewise linear regression is automatically determined using the "piecewise.linear" function, and the 97.5% confidence interval is estimated using 1000 bootstraps.
> model <- piecewise.linear(x = df1$$R_h$, y = df1$ CUE_{ST},
CI = TRUE, bootstrap.samples = 1000, sig.level = 0.05)
> model
Threshold alpha = **340.31**

CI	Change point	Initial slope	Slope change	Second slope
2.50%	**321.5715**	-0.00127801	0.000916009	-2.79e-05
97.50%	**356.584**	-0.000908321	0.001311988	6.35e-05

Table S5:

Execution and results of the piecewise regression analyses for identifying relationships between microbial C use efficiency (CUE_{ST}) and annual heterotrophic respiration rate (R_h, g C kg^{-1} soil y^{-1}) (corresponding to Supplemental Fig. S6A).

First step:

Calculate the adjusted R^2 and p of the piecewise linear regression using the "segmented" package

#Fitting a simple linear regression model between CUE_{ST} and R_h_BD first
fit_lm <- lm(CUE_{ST} ~R_h_BD, data = df1)
summary(fit_lm)

#In the above known linear regression model, the possible breakpoints were found by the "segmented" function.
> lm_seg1 <- segmented(fit_lm, seg.Z = ~ R_h_BD, npsi = 1)
> summary(lm_seg1)
Call:
segmented.lm(obj = fit_lm, seg.Z = ~ R_h_BD, npsi = 1)

Estimated breakpoint(s):

	Est.	St.Err
psi1. R_h_BD:	**2630.403**	79.357

Meaningful coefficients of the linear terms:

	Estimate	Std. Error	*t*	Pr(>|t|)	Significance
(Intercept)	6.173e-01	2.365e-02	26.10	<2e-16	***
R_h_BD	-1.403e-04	1.153e-05	-12.18	<2e-16	***
U1.R_h_BD	1.391e-04	1.168e-05	11.91	NA	

***, $P < 0.001$
Residual standard error: 0.09693 on 1090 degrees of freedom
Multiple R^2 = 0.2494, adjusted R^2 = **0.2473**

Boot restarting based on six samples. Last fit:
Convergence attained in two iterations (rel. change 1.2344e-11)

Second step:

Calculate the confidence interval of piecewise linear regression using the "SiZer" package

#The breakpoint of the piecewise linear regression is automatically determined using the "piecewise.linear" function, and the 97.5% confidence interval is estimated using 1000 bootstraps.
> model <- piecewise.linear(x = df1$$R_h$_BD, y = df1$ CUE_{ST},
CI = TRUE, bootstrap.samples = 1000, sig.level = 0.05)
> model
Threshold alpha = **2630.4027655103**

CI	Change point	Initial slope	Slope change	Second slope
2.50%	**2412.147**	-0.0001762985	0.000115545	-4.573952e-06
97.50%	**2778.070**	-0.0001176477	0.000174432	1.740343e-06

Table S6:

Execution and results of the piecewise regression analyses for identifying relationships between microbial C use efficiency (CUE_{ST}) and average daily heterotrophic respiration rate (average daily R_h, g C m^{-2} soil d^{-1}) (corresponding to Fig. 3C).

First step:

Calculate the adjusted R^2 and *p* of the piecewise linear regression using the "segmented" package

#Fitting a simple linear regression model between CUE_{ST} and average daily R_h first
fit_lm <- lm(CUE_{ST} ~daily_R_h, data = df1)
summary(fit_lm)

#In the above known linear regression model, the possible breakpoints were found by the "segmented" function.
> lm_seg1 <- segmented(fit_lm, seg.Z = ~ daily_R_h, npsi = 1)
> summary(lm_seg1)
Call:
segmented.lm(obj = fit_lm, seg.Z = ~ R_h, npsi = 1)

Estimated breakpoint(s):

	Est.	St.Err
psi1. daily_R_h:	**5.965**	1.437

Meaningful coefficients of the linear terms:

	Estimate	Std. Error	*t*	Pr(>|t|)	Significance
(Intercept)	0.404556	0.027476	14.724	<2e-16	***
daily_R_h	-0.033973	0.008383	-4.052	0.000185	***
U1.daily_R_h	0.035276	0.011022	3.200	NA	

***, $P < 0.001$
Residual standard error: 0.09173 on 48 degrees of freedom
Multiple R^2 = 0.3853, adjusted R^2 = **0.3469**

Boot restarting based on eight samples. Last fit:
Convergence attained in two iterations (rel. change 1.1789e-08)

Second step:

Calculate the confidence interval of piecewise linear regression using the "SiZer" package

#The breakpoint of the piecewise linear regression is automatically determined using the "piecewise.linear" function, and the 97.5% confidence interval is estimated using 1000 bootstraps.
> model <- piecewise.linear(x = df1$daily_$R_h$, y = df1$ CUE_{ST},
CI = TRUE, bootstrap.samples = 1000, sig.level = 0.05)
> model
Threshold alpha = **5.964566**

CI	Change point	Initial slope	Slope change	Second slope
2.50%	**2.664985**	-0.08051737	0.01687438	-0.007942019
97.50%	**8.396705**	-0.01940728	0.07961223	0.015567094

Table S7

Normality tests for log-transformed CUE_{ST}/R_h by productivity (low *vs* high productivity ecosystems).

Productivity	*n*	shapiro_*W*	shapiro_*p*	lillie_*D*	lillie_*p*	ad_*A*	ad_*p*
Low	612	0.967	**1.68e-10**	0.104	**1.57e-17**	7.4	**4.21e-18**
High	482	0.938	**3.07e-13**	0.119	**5.49e-18**	10.5	**3.7 e-24**

Note: Reported are sample size (*n*), Shapiro-Wilk *W*, Lilliefors-corrected KS *D*, Anderson–Darling *A*, and *p*-values. Values indicate significant departures from normality in both productivity groups ($p < 0.001$).

Table S8

OLS estimates for log-transformed CUE_{ST}/R_h with HC3 robust standard errors by productivity (low *vs* high productivity ecosystems).

	Low productivity					High productivity				
Breusch-Pagan test	BP = 43.46, df = 9, *p*-value = 1.775e-06					BP = 99.581, df = 9, *p*-value < 2.2e-16				
	Estimate	Std. Error	t value	Pr(>|t|)		Estimate	Std. Error	t value	Pr(>|t|)	
(Intercept)	-2.719	0.017	-160.125	< 2.2e-16	***	-3.158	0.020	-156.498	< 2.2e-16	***
MAP	-0.416	0.050	-8.372	3.98e-16	***	-0.196	0.052	-3.749	1.995e-04	***
LAI	-0.114	0.021	-5.377	1.088e-07	***	0.026	0.026	0.990	0.323	
Soil MAT	0.431	0.127	3.391	7.43e-04	***	0.665	0.124	5.376	1.202e-07	***
Soil pH	0.050	0.023	2.153	0.032	*	-0.099	0.025	-3.950	9.016e-05	***
MAT	-0.706	0.151	-4.673	3.673e-06	***	-0.833	0.162	-5.152	3.803e-07	***
AGB	0.144	0.043	3.316	9.70e-04	***	-0.099	0.173	-0.574	0.566	
BGB	-0.216	0.041	-5.240	2.225e-07	***	0.087	0.177	0.494	0.621	
Clay	0.091	0.027	3.415	6.802e-04	***	0.052	0.027	1.936	0.0535	.
CEC	-0.001	0.025	-0.031	0.976		-0.072	0.039	-1.827	0.0683	.

Signif. codes: 0 ‘***’ 0.001 ‘**’ 0.01 ‘*’ 0.05 ‘.’ 0.1 ‘ ’ 1

Note: Predictors were standardized (mean 0, SD 1). Entries show coefficient estimates, HC3 *SE*, *t* and *p*. Breusch-Pagan tests indicated heteroscedasticity in both productivity groups (Low: $p < 0.001$; High: $p < 0.001$). For interpretation, coefficients can be approximated as percent changes via 100 × (exp(β)-1) per 1 SD increase in the predictor.

Table S9

Gamma generalized linear models (log link) for CUE_{ST}/R_h by productivity (low *vs* high productivity ecosystems).

	Low productivity					High productivity				
	Estimate	Std. Error	*t* value	Pr(>|t|)		Estimate	Std. Error	*t* value	Pr(>|t|)	
(Intercept)	-1.580	0.116	-13.584	< 2e-16	***	-1.471	0.276	-5.339	1.45e-07	***
MAP	0.000	0.000	-10.619	< 2e-16	***	0.000	0.000	-3.862	1.28e-04	***
LAI	-0.115	0.021	-5.475	6.42e-08	***	0.046	0.042	1.082	0.280	
Soil MAT	0.047	0.010	4.709	3.10e-06	***	0.077	0.014	5.619	3.29e-08	***
Soil pH	0.016	0.013	1.248	2.12e-01		-0.105	0.023	-4.562	6.47e-06	***
MAT	-0.071	0.010	-7.280	1.05e-12	***	-0.102	0.014	-7.184	2.66e-12	***
AGB	0.000	0.000	3.106	0.002	**	0.000	0.000	0.151	0.880	
BGB	-0.003	0.001	-5.063	5.51e-07	***	0.000	0.001	-0.339	0.734	
Clay	0.006	0.002	3.329	9.26e-04	***	0.004	0.003	1.522	0.129	
CEC	0.000	0.000	0.239	0.811041		0.000	0.000	-2.719	0.007	**
	(Dispersion parameter for Gamma family taken to be 0.142)<br>Null deviance: 443.171 on 611 degrees of freedom<br>Residual deviance: 95.016 on 602 degrees of freedom<br>AIC: -2693.8					(Dispersion parameter for Gamma family taken to be 0.212)<br>Null deviance: 184.579 on 481 degrees of freedom<br>Residual deviance: 90.332 on 472 degrees of freedom<br>AIC: -2446.3				

Signif. codes: 0 '***' 0.001 '**' 0.01 '*' 0.05 '.' 0.1 ' ' 1

Note: Reported are coefficients, standard errors, *t*-values, *p*-values, model AIC, and dispersion. Signs and significance patterns are consistent with HC3-robust OLS, indicating robustness to distributional assumptions. Under the log link, exp(β)-1 approximates the percent change in the mean response per 1 SD increase in the predictor.

Table S10

Description of environmental variables used as the potential drivers in affecting both microbial C use efficiency (CUE_{ST}) and average annual heterotrophic respiration rate (R_h).

Resource types	Abbreviation	Variables	Unit	Sources	Period/layer	Reference
Temperature	MAT	Mean annual temperature	°C	WorldClim2	1970-2000	*73*
	Soil MAT	Soil mean annual temperature	°C	https://zenodo.org/record/4558732#.Y2N9XsiUdvJ	1979-2013/0-5cm	*56*
Water	MAP	Mean annual precipitation	mm	WorldClim2	1970-2000	*73*
Carbon source	LAI	Leaf area index	m^2 m^{-2}	https://daac.ornl.gov/cgi-bin/dsviewer.pl?ds_id=1653	1981-2015	*74*
	AGB	Aboveground biomass	Mg C ha^{-1}	https://daac.ornl.gov/VEGETATION/guides/Global_Maps_C_Density_2010.html	2010	*75*
	BGB	Belowground biomass	Mg C ha^{-1}	https://daac.ornl.gov/VEGETATION/guides/Global_Maps_C_Density_2010.html	2010	*75*
Nutrients	pH	Soil pH	-	Collected by this study	1980-2022	See the attached database
	CEC	Cation exchange capacity	cmol kg^{-1}	http://globalchange.bnu.edu.cn/research/soilw#download	0-10 cm	*76*
	Clay	Soil clay content	% of weight	http://globalchange.bnu.edu.cn/research/soilw#download	0-10 cm	*76*
-	BD	Soil bulk density	g cm^{-3}	http://globalchange.bnu.edu.cn/research/soilw#download	0-10 cm	*76*

REFERENCES

1. Y. Kuzyakov, Sources of CO_2 efflux from soil and review of partitioning methods. *Soil Biol. Biochem.* **38**, 425–448 (2006).

2. B. Bond-Lamberty, V. L. Bailey, M. Chen, C. M. Gough, R. Vargas, Globally rising soil heterotrophic respiration over recent decades. *Nature* **560**, 80–83 (2018).

3. J. Schimel, M. N. Weintraub, D. L. Moorhead, Estimating microbial carbon use efficiency in soil: Isotope-based and enzyme-based methods measure fundamentally different aspects of microbial resource use. *Soil Biol. Biochem.* **169**, 108677 (2022).

4. S. Manzoni, P. Taylor, A. Richter, A. Porporato, G. I. Ågren, Environmental and stoichiometric controls on microbial carbon-use efficiency in soils. *New Phytol.* **196**, 79–91 (2012).

5. M. F. Cotrufo, J. L. Soong, A. J. Horton, E. E. Campbell, M. L. Haddix, D. H. Wall, W. J. Parton, Formation of soil organic matter via biochemical and physical pathways of litter mass loss. *Nat. Geosci.* **8**, 776–779 (2015).

6. F. Tao, Y. Huang, B. A. Hungate, S. Manzoni, S. D. Frey, M. W. Schmidt, M. Reichstein, N. Carvalhais, P. Ciais, L. Jiang, J. Lehmann, Y. Wang, B. Z. Houlton, B. Ahrens, U. Mishra, G. Hugelius, T. D. Hocking, X. Lu, Z. Shi, K. Viatkin, R. Vargas, Y. Yigini, C. Omuto, A. A. Malik, G. Peralta, R. Cuevas-Corona, L. E. Di Paolo, I. Luotto, C. Liao, Y. Liang, V. S. Saynes, X. Huang, Y. Luo, Microbial carbon use efficiency promotes global soil carbon storage. *Nature* **618**, 981–985 (2023).

7. S. B. Hagerty, K. J. Van Groenigen, S. D. Allison, B. A. Hungate, E. Schwartz, G. W. Koch, R. K. Kolka, P. Dijkstra, Accelerated microbial turnover but constant growth efficiency with warming in soil. *Nat. Clim. Change* **4**, 903–906 (2014).

8. T. P. Smith, T. Clegg, T. Bell, S. Pawar, Systematic variation in the temperature dependence of bacterial carbon use efficiency. *Ecol. Lett.* **24**, 2123–2133 (2021).

9. A. Nissan, U. Alcolombri, N. Peleg, N. Galili, J. Jimenez-Martinez, P. Molnar, M. Holzner, Global warming accelerates soil heterotrophic respiration. *Nat. Commun.* **14**, 3452 (2023).

10. C. Yue, J. Jian, P. Ciais, X. Ren, J. Jiao, S. An, Y. Li, J. Wu, P. Zhang, B. Bond-Lamberty, Field experiments show no consistent reductions in soil microbial carbon in response to warming. *Nat. Commun.* **15**, 1731 (2024).

11. J. Schroeder, C. Dămătîrcă, T. Bölscher, C. Chenu, L. Elsgaard, C. C. Tebbe, L. Skadell, C. Poeplau, Liming effects on microbial carbon use efficiency and its potential consequences for soil organic carbon stocks. *Soil Biol. Biochem.* **191**, 109342 (2024).

12. L. Fang, Multifaceted links between microbial carbon use efficiency and soil organic carbon sequestration. *Glob. Chang. Biol.* **31**, e70045 (2025).

13. R. G. Burns, R. P. Dick, *Enzymes in the Environment: Activity, Ecology, and Applications* (CRC Press, 2002).

14. C. Wang, Y. Kuzyakov, Energy use efficiency of soil microorganisms: Driven by carbon recycling and reduction. *Glob. Chang. Biol.* **29**, 6170–6187 (2023).

15. L. Qu, C. Wang, E. Bai, Evaluation of the ^{18}O-H_2O incubation method for measurement of soil microbial carbon use efficiency. *Soil Biol. Biochem.* **145**, 107802 (2020).

16. C. Wang, L. Qu, L. Yang, D. Liu, E. Morrissey, R. Miao, Z. Liu, Q. Wang, Y. Fang, E. Bai, Large-scale importance of microbial carbon use efficiency and necromass to soil organic carbon. *Glob. Change Biol.* **27**, 2039–2048 (2021).

17. J. Hu, C. Huang, S. Zhou, Y. Kuzyakov, Nitrogen addition to soil affects microbial carbon use efficiency: Meta-analysis of similarities and differences in ^{13}C and ^{18}O approaches. *Glob. Change Biol.* **28**, 4977–4988 (2022).

18. K. Yu, L. He, S. Niu, J. Wang, P. Garcia-Palacios, M. Dacal, C. Averill, K. Georgiou, J. Ye, F. Mo, L. Yang, T. W. Crowther, Nonlinear microbial thermal response and its implications for abrupt soil organic carbon responses to warming. *Nat. Commun.* **16**, 2763 (2025).

19. H. Doi, M. Cherif, T. Iwabuchi, I. Katano, J. C. Stegen, M. Striebel, Integrating elements and energy through the metabolic dependencies of gross growth efficiency and the threshold elemental ratio. *Oikos* **119**, 752–765 (2010).

20. J. E. Hobbie, E. A. Hobbie, Microorganisms in nature are limited by carbon and energy: The starving-survival lifestyle in soil and consequences for estimating microbial rates. *Front. Microbiol.* **4**, 324 (2013).

21. J. Chen, K. J. van Groenigen, B. A. Hungate, C. Terrer, J. W. van Groenigen, F. T. Maestre, S. C. Ying, Y. Luo, U. Jørgensen, R. L. Sinsabaugh, J. E. Olesen, L. Elsgaard, Long-term nitrogen loading alleviates phosphorus limitation in terrestrial ecosystems. *Glob. Change Biol.* **26**, 5077–5086 (2020).

22. Y. Cui, D. L. Moorhead, X. Wang, M. Xu, X. Wang, X. Wei, Z. Zhu, T. Ge, S. Peng, B. Zhu, L. Fang, Decreasing microbial phosphorus limitation increases soil carbon release. *Geoderma* **419**, 115868 (2022).

23. C. Averill, Divergence in plant and microbial allocation strategies explains continental patterns in microbial allocation and biogeochemical fluxes. *Ecol. Lett.* **17**, 1202–1210 (2014).

24. R. L. Sinsabaugh, J. J. Follstad Shah, Ecoenzymatic stoichiometry and ecological theory. *Annu. Rev. Ecol. Evol. Syst.* **43**, 313–343 (2012).

25. J. Jian, R. Vargas, K. Anderson-Teixeira, E. Stell, V. Herrmann, M. Horn, N. Kholod, J. Manzon, R. Marchesi, D. Paredes, B. Bond-Lamberty, A restructured and updated global soil respiration database (SRDB-V5). *Earth Syst. Sci. Data* **13**, 255–267 (2021).

26. L. Henneron, J. Balesdent, G. Alvarez, P. Barré, F. Baudin, I. Basile-Doelsch, L. Cécillon, A. Fernandez-Martinez, C. Hatté, S. Fontaine, Bioenergetic control of soil carbon dynamics across depth. *Nat. Commun.* **13**, 7676 (2022).

27. A. Hursh, A. Ballantyne, L. Cooper, M. Maneta, J. Kimball, J. Watts, The sensitivity of soil respiration to soil temperature, moisture, and carbon supply at the global scale. *Glob. Change Biol.* **23**, 2090–2103 (2017).

28. E. Du, C. Terrer, A. F. Pellegrini, A. Ahlström, C. J. van Lissa, X. Zhao, N. Xia, X. Wu, R. B. Jackson, Global patterns of terrestrial nitrogen and phosphorus limitation. *Nat. Geosci.* **13**, 221–226 (2020).

29. Y. Cui, H. Bing, D. L. Moorhead, M. Delgado-Baquerizo, L. Ye, J. Yu, S. Zhang, X. Wang, S. Peng, X. Guo, B. Zhu, J. Chen, W. Tan, Y. Wang, X. Zhang, L. Fang, Ecoenzymatic stoichiometry reveals widespread soil phosphorus limitation to microbial metabolism across Chinese forests. *Commun. Earth Environ.* **3**, 1–8 (2022).

30. Y. Cui, S. Peng, M. C. Rillig, T. Camenzind, M. Delgado-Baquerizo, C. Terrer, X. Xu, M. Feng, M. Wang, L. Fang, B. Zhu, E. Du, D. L. Moorhead, R. L. Sinsabaugh, J. Peñuelas, J. J. Elser, Global patterns of nutrient limitation in soil microorganisms. *Proc. Natl. Acad. Sci. U.S.A.* **122**, e2424552122 (2025).

31. A. T. Nottingham, B. L. Turner, A. W. Stott, E. V. Tanner, Nitrogen and phosphorus constrain labile and stable carbon turnover in lowland tropical forest soils. *Soil Biol. Biochem.* **80**, 26–33 (2015).

32. J. Helfenstein, F. Tamburini, C. von Sperber, M. S. Massey, C. Pistocchi, O. A. Chadwick, P. M. Vitousek, R. Kretzschmar, E. Frossard, Combining spectroscopic and isotopic techniques gives a dynamic view of phosphorus cycling in soil. *Nat. Commun.* **9**, 3226 (2018).

33. M. F. Cotrufo, M. D. Wallenstein, C. M. Boot, K. Denef, E. Paul, The Microbial Efficiency-Matrix Stabilization (MEMS) framework integrates plant litter decomposition with soil organic matter stabilization: do labile plant inputs form stable soil organic matter? *Glob. Change Biol.* **19**, 988–995 (2013).

34. E. M. Morrissey, R. L. Mau, E. Schwartz, T. A. McHugh, P. Dijkstra, B. J. Koch, J. C. Marks, B. A. Hungate, Bacterial carbon use plasticity, phylogenetic diversity and the priming of soil organic matter. *ISME J.* **11**, 1890–1899 (2017).

35. M. A. Bradford, R. L. McCulley, T. W. Crowther, E. E. Oldfield, S. A. Wood, N. Fierer, Cross-biome patterns in soil microbial respiration predictable from evolutionary theory on thermal adaptation. *Nat. Ecol. Evol.* **3**, 223–231 (2019).

36. J. Lehmann, M. Kleber, The contentious nature of soil organic matter. *Nature* **528**, 60–68 (2015).

37. T. Camenzind, S. Hättenschwiler, K. K. Treseder, A. Lehmann, M. C. Rillig, Nutrient limitation of soil microbial processes in tropical forests. *Ecol. Monogr.* **88**, 4–21 (2018).

38. X. Fan, E. Bai, J. Zhang, X. Wang, W. Yuan, S. Piao, The carbon transfer from plant to soil is more efficient in less productive ecosystems. *Global Biogeochem. Cy.* **37**, e2023GB007727 (2023).

39. A. P. Allen, J. F. Gillooly, Towards an integration of ecological stoichiometry and the metabolic theory of ecology to better understand nutrient cycling. *Ecol. Lett.* **12**, 369–384 (2009).

40. S. Manzoni, A. Porporato, Soil carbon and nitrogen mineralization: Theory and models across scales. *Soil Biol. Biochem.* **41**, 1355–1379 (2009).

41. Z. Zhu, S. Piao, R. B. Myneni, M. Huang, Z. Zeng, J. G. Canadell, P. Ciais, S. Sitch, P. Friedlingstein, A. Arneth, C. Cao, L. Cheng, E. Kato, C. Koven, Y. Li, X. Lian, Y. Liu, R. Liu, J. Mao, Y. Pan, S. Peng, J. Peñuelas, B. Poulter, T. A. M. Pugh, B. D. Stocker, N. Viovy, X. Wang, Y. Wang, Z. Xiao, H. Yang, S. Zaehle, N. Zeng, Greening of the Earth and its drivers. *Nat. Clim. Change* **6**, 791–795 (2016).

42. IPCC, *Climate Change 2022: Impacts, Adaptation and Vulnerability. IPCC Sixth Assessment Report* (IPCC, 2022).

43. Y. Wang, J. Xiao, Y. Ma, J. Ding, X. Chen, Z. Ding, Y. Luo, Persistent and enhanced carbon sequestration capacity of alpine grasslands on Earth's Third Pole. *Sci. Adv.* **9**, eade6875 (2023).

44. S. Piao, Q. Liu, A. Chen, I. A. Janssens, Y. Fu, J. Dai, L. Liu, X. Lian, M. Shen, X. Zhu, Plant phenology and global climate change: Current progresses and challenges. *Glob. Change Biol.* **25**, 1922–1940 (2019).

45. J. Tian, J. A. Dungait, X. Lu, Y. Yang, I. P. Hartley, W. Zhang, J. Mo, G. Yu, J. Zhou, Y. Kuzyakov, Long-term nitrogen addition modifies microbial composition and functions for

slow carbon cycling and increased sequestration in tropical forest soil. *Glob. Chang. Biol.* **25**, 3267–3281 (2019).

46. Z. Mou, L. Kuang, J. Zhang, Y. Li, W. Wu, C. Liang, D. Hui, H. Lambers, J. Sardans, J. Peñuelas, J. Liu, H. Ren, Z. Liu, Nutrient availability and stoichiometry mediate microbial effects on soil carbon sequestration in tropical forests. *Soil Biol. Biochem.* **186**, 109186 (2023).

47. J. Penuelas, B. Poulter, J. Sardans, P. Ciais, M. Van Der Velde, L. Bopp, O. Boucher, Y. Godderis, P. Hinsinger, J. Llusia, E. Nardin, S. Vicca, M. Obersteiner, I. A. Janssens, Human-induced nitrogen-phosphorus imbalances alter natural and managed ecosystems across the globe. *Nat. Commun.* **4**, 2934 (2013).

48. G. Yu, Y. Jia, N. He, J. Zhu, Z. Chen, Q. Wang, S. Piao, X. Liu, H. He, X. Guo, Z. Wen, P. Li, G. Ding, K. Goulding, Stabilization of atmospheric nitrogen deposition in China over the past decade. *Nat. Geosci.* **12**, 424–429 (2019).

49. D. L. Jones, E. C. Cooledge, F. C. Hoyle, R. I. Griffiths, D. V. Murphy, pH and exchangeable aluminum are major regulators of microbial energy flow and carbon use efficiency in soil microbial communities. *Soil Biol. Biochem.* **138**, 107584 (2019).

50. C. L. Lauber, M. Hamady, R. Knight, N. Fierer, Pyrosequencing-based assessment of soil pH as a predictor of soil bacterial community structure at the continental scale. *Appl. Environ. Microbio.* **75**, 5111–5120 (2009).

51. P. Čapek, S. Manzoni, E. Kaštovská, B. Wild, K. Diáková, J. Bárta, J. Schnecker, C. Biasi, P. J. Martikainen, R. J. Eloy Alves, G. Guggenberger, N. Gentsch, G. Hugelius, J. Palmtag, R. Mikutta, O. Shibistova, T. Urich, C. Schleper, A. Richter, H. Šantrůčková, A plant-microbe interaction framework explaining nutrient effects on primary production. *Nat. Ecol. Evol.* **2**, 1588–1596 (2018).

52. J. L. DeForest, D. L. Moorhead, Effects of elevated pH and phosphorus fertilizer on soil C, N and P enzyme stoichiometry in an acidic mixed mesophytic deciduous forest. *Soil Biol. Biochem.* **150**, 107996 (2020).

53. Y. Cui, J. Hu, S. Peng, M. Delgado-Baquerizo, D. L. Moorhead, R. L. Sinsabaugh, X. Xu, K. M. Geyer, L. Fang, P. Smith, J. Peñuelas, Y. Kuzyakov, J. Chen, Limiting resources define the global pattern of soil microbial carbon use efficiency. *Adv. Sci.* **11**, e2308176 (2024).

54. T. W. Crowther, J. Van den Hoogen, J. Wan, M. A. Mayes, A. D. Keiser, L. Mo, C. Averill, D. S. Maynard, The global soil community and its influence on biogeochemistry. *Science* **365**, eaav0550 (2019).

55. L. Philippot, C. Chenu, A. Kappler, M. C. Rillig, N. Fierer, The interplay between microbial communities and soil properties. *Nat. Rev. Microbiol.* **22**, 226–239 (2024).

56. J. J. Lembrechts, J. van den Hoogen, J. Aalto, M. B. Ashcroft, P. De Frenne, J. Kemppinen, M. Kopecký, M. Luoto, I. M. D. Maclean, T. W. Crowther, J. J. Bailey, S. Haesen, D. H. Klinges, P. Niittynen, B. R. Scheffers, K. Van Meerbeek, P. Aartsma, O. Abdalaze, M. Abedi, R. Aerts, N. Ahmadian, A. Ahrends, J. M. Alatalo, J. M. Alexander, C. N. Allonsius, J. Altman, C. Ammann, C. Andres, C. Andrews, J. Ardö, N. Arriga, A. Arzac, V. Aschero, R. L. Assis, J. J. Assmann, M. Y. Bader, K. Bahalkeh, P. Barančok, I. C. Barrio, A. Barros, M. Barthel, E. W. Basham, M. Bauters, M. Bazzichetto, L. B. Marchesini, M. C. Bell, J. C. Benavides, J. L. B. Alonso, B. J. Berauer, J. W. Bjerke, R. G. Björk, M. P. Björkman, K. Björnsdóttir, B. Blonder, P. Boeckx, J. Boike, S. Bokhorst, B. N. S. Brum, J. Brůna, N. Buchmann, P. Buysse, J. L. Camargo, O. C. Campoe, O. Candan, R. Canessa, N. Cannone, M. Carbognani, J. Carnicer, A. Casanova-Katny, S. Cesarz, B. Chojnicki, P. Choler, S. L. Chown, E. F. Cifuentes, M. Čiliak, T. Contador, P. Convey, E. J. Cooper, E. Cremonese, S. R. Curasi, R. Curtis, M. Cutini, C. J. Dahlberg, G. N. Daskalova, M. A. de Pablo, S. D. Chiesa, J. Dengler, B. Deronde, P. Descombes, V. D. Cecco, M. D. Musciano, J. Dick, R. D. Dimarco, J. Dolezal, E. Dorrepaal, J. Dušek, N. Eisenhauer, L. Eklundh, T. E. Erickson, B. Erschbamer, W. Eugster, R. M. Ewers, D. A. Exton, N. Fanin, F. Fazlioglu, I. Feigenwinter, G. Fenu, O. Ferlian, M. R. F. Calzado, E. Fernández-Pascual, M. Finckh, R. F. Higgens, T. G. W. Forte, E. C. Freeman, E. R. Frei, E. Fuentes-Lillo, R. A. García, M. B. García, C. Géron, M. Gharun, D. Ghosn, K. Gigauri, A. Gobin, I. Goded, M. Goeckede, F. Gottschall, K. Goulding, S. Govaert, B. J. Graae, S. Greenwood, C. Greiser, A. Grelle, B. Guénard, M. Guglielmin, J. Guillemot, P. Haase, S. Haider, A. H. Halbritter, M. Hamid, A. Hammerle, A. Hampe, S. V. Haugum, L. Hederová, B. Heinesch, C. Helfter, D. Hepenstrick, M. Herberich,

M. Herbst, L. Hermanutz, D. S. Hik, R. Hoffrén, J. Homeier, L. Hörtnagl, T. T. Høye, F. Hrbacek, K. Hylander, H. Iwata, M. A. Jackowicz-Korczynski, H. Jactel, J. Järveoja, S. Jastrzębowski, A. Jentsch, J. J. Jiménez, I. S. Jónsdóttir, T. Jucker, A. S. Jump, R. Juszczak, R. Kanka, V. Kašpar, G. Kazakis, J. Kelly, A. A. Khuroo, L. Klemedtsson, M. Klisz, N. Kljun, A. Knohl, J. Kobler, J. Kollár, M. M. Kotowska, B. Kovács, J. Kreyling, A. Lamprecht, S. I. Lang, C. Larson, K. Larson, K. Laska, G. le Maire, R. I. Leihy, L. Lens, B. Liljebladh, A. Lohila, J. Lorite, B. Loubet, J. Lynn, M. Macek, R. Mackenzie, E. Magliulo, R. Maier, F. Malfasi, F. Máliš, M. Man, G. Manca, A. Manco, T. Manise, P. Manolaki, F. Marciniak, R. Matula, A. C. Mazzolari, S. Medinets, V. Medinets, C. Meeussen, S. Merinero, R. de Cássia Guimarães Mesquita, K. Meusburger, F. J. R. Meysman, S. T. Michaletz, A. Milbau, D. Moiseev, P. Moiseev, A. Mondoni, R. Monfries, L. Montagnani, M. Moriana-Armendariz, U. M. di Cella, M. Mörsdorf, J. R. Mosedale, L. Muffler, M. Muñoz-Rojas, J. A. Myers, I. H. Myers-Smith, L. Nagy, M. Nardino, I. Naujokaitis-Lewis, E. Newling, L. Nicklas, G. Niedrist, A. Niessner, M. B. Nilsson, S. Normand, M. D. Nosetto, Y. Nouvellon, M. A. Nuñez, R. Ogaya, J. Ogée, J. Okello, J. Olejnik, J. E. Olesen, Ø. H. Opedal, S. Orsenigo, A. Palaj, T. Pampuch, A. V. Panov, M. Pärtel, A. Pastor, A. Pauchard, H. Pauli, M. Pavelka, W. D. Pearse, M. Peichl, L. Pellissier, R. M. Penczykowski, J. Penuelas, M. P. Bon, A. Petraglia, S. S. Phartyal, G. K. Phoenix, C. Pio, A. Pitacco, C. Pitteloud, R. Plichta, F. Porro, M. Portillo-Estrada, J. Poulenard, R. Poyatos, A. S. Prokushkin, R. Puchalka, M. Pușcaș, D. Radujković, K. Randall, A. R. Backes, S. Remmele, W. Remmers, D. Renault, A. C. Risch, C. Rixen, S. A. Robinson, B. J. M. Robroek, A. V. Rocha, C. Rossi, G. Rossi, O. Roupsard, A. V. Rubtsov, P. Saccone, C. Sagot, J. S. Bravo, C. C. Santos, J. M. Sarneel, T. Scharnweber, J. Schmeddes, M. Schmidt, T. Scholten, M. Schuchardt, N. Schwartz, T. Scott, J. Seeber, A. C. S. de Andrade, T. Seipel, P. Semenchuk, R. A. Senior, J. M. Serra-Diaz, P. Sewerniak, A. Shekhar, N. V. Sidenko, L. Siebicke, L. S. Collier, E. Simpson, D. P. Siqueira, Z. Sitková, J. Six, M. Smiljanic, S. W. Smith, S. Smith-Tripp, B. Somers, M. V. Sørensen, J. J. L. L. Souza, B. I. Souza, A. S. Dias, M. J. Spasojevic, J. D. M. Speed, F. Spicher, A. Stanisci, K. Steinbauer, R. Steinbrecher, M. Steinwandter, M. Stemkovski, J. G. Stephan, C. Stiegler, S. Stoll, M. Svátek, M. Svoboda, T. Tagesson, A. J. Tanentzap, F. Tanneberger, J.-P. Theurillat, H. J. D. Thomas, A. D. Thomas, K. Tielbörger, M. Tomaselli, U. A. Treier, M. Trouillier, P. D. Turtureanu, R. Tutton, V. A. Tyystjärvi, M. Ueyama, K. Ujházy, M. Ujházyová, D. Uogintas, A. V. Urban, J. Urban, M. Urbaniak, T.-M. Ursu, F. P. Vaccari, S.

Van de Vondel, L. van den Brink, M. Van Geel, V. Vandvik, P. Vangansbeke, A. Varlagin, G. F. Veen, E. Veenendaal, S. E. Venn, H. Verbeeck, E. Verbrugggen, F. G. A. Verheijen, L. Villar, L. Vitale, P. Vittoz, M. Vives-Ingla, J. von Oppen, J. Walz, R. Wang, Y. Wang, R. G. Way, R. E. M. Wedegärtner, R. Weigel, J. Wild, M. Wilkinson, M. Wilmking, L. Wingate, M. Winkler, S. Wipf, G. Wohlfahrt, G. Xenakis, Y. Yang, Z. Yu, K. Yu, F. Zellweger, J. Zhang, Z. Zhang, P. Zhao, K. Ziemblińska, R. Zimmermann, S. Zong, V. I. Zyryanov, I. Nijs, J. Lenoir, Global maps of soil temperature. *Glob. Change Biol.* **28**, 3110–3144 (2022).

57. Y. Cui, S. Peng, M. Delgado-Baquerizo, M. C. Rillig, C. Terrer, B. Zhu, X. Jing, J. Chen, J. Li, J. Feng, Y. He, L. Fang, D. L. Moorhead, R. L. Sinsabaugh, J. Peñuelas, Microbial communities in terrestrial surface soils are not widely limited by carbon. *Glob. Chang. Biol.* **29**, 4412–4429 (2023).

58. D. Curtin, C. A. Campbell, A. Jalil, Effects of acidity on mineralization: pH-dependence of organic matter mineralization in weakly acidic soils. *Soil Biol. Biochem.* **30**, 57–64 (1998).

59. N. J. Barrow, Comparing two theories about the nature of soil phosphate. *Eur. J. Soil Syst.* **72**, 679–685 (2021).

60. R. L. Sinsabaugh, B. H. Hill, J. J. Follstad Shah, Ecoenzymatic stoichiometry of microbial organic nutrient acquisition in soil and sediment. *Nature* **462**, 795–798 (2009).

61. D. L. Moorhead, R. L. Sinsabaugh, A theoretical model of litter decay and microbial interaction. *Ecol. Monogr.* **76**, 151–174 (2006).

62. D. L. Moorhead, Y. Cui, R. L. Sinsabaugh, J. Schimel, Interpreting patterns of ecoenzymatic stoichiometry. *Soil Biol. Biochem.* **180**, 108997 (2023).

63. M. F. Cotrufo, J. M. Lavallee, Soil organic matter formation, persistence, and functioning: A synthesis of current understanding to inform its conservation and regeneration. *Adv. Agron.* **172**, 1–66 (2022).

64. H. E. Beck, N. E. Zimmermann, T. R. McVicar, N. Vergopolan, A. Berg, E. F. Wood, Present and future Köppen-Geiger climate classification maps at 1-km resolution. *Sci. Data* **5**, 1–12 (2018).

65. V. M. R. Muggeo, Segmented: An R package to fit regression models with broken-line relationships. *R News* **8**, 20–25 (2008).

66. J. D. Toms, M. Lesperance, Piecewise regression: A tool for identifying ecological thresholds. *Ecology* **84**, 2034–2041 (2003).

67. V. Calcagno, C. de Mazancourt, glmulti: An R package for easy automated model selection with (generalized) linear models. *J. Stat. Softw.* **34**, 1–29 (2010).

68. R Core Team, "R: A language and environment for statistical computing" (R Foundation for Statistical Computing, 2024); www.R-project.org/.

69. R. L. Sinsabaugh, B. L. Turner, J. M. Talbot, B. G. Waring, J. S. Powers, C. R. Kuske, D. L. Moorhead, J. J. Follstad Shah, Stoichiometry of microbial carbon use efficiency in soils. *Ecol. Monogr.* **86**, 172–189 (2016).

70. S. Manzoni, Flexible carbon-use efficiency across litter types and during decomposition partly compensates nutrient imbalances-results from analytical stoichiometric models. *Front. Microbiol.* **8**, 661 (2017).

71. S. Manzoni, P. Čapek, M. Mooshammer, B. D. Lindahl, A. Richter, H. Šantrůčková, Optimal metabolic regulation along resource stoichiometry gradients. *Ecol. Lett.* **20**, 1182–1191 (2017).

72. A. Gunina, Y. Kuzyakov, From energy to (soil organic) matter. *Glob. Change Biol.* **28**, 2169–2182 (2022).

73. S. E. Fick, R. J. Hijmans, WorldClim 2: New 1km spatial resolution climate surfaces for global land areas. *Int. J. Climatol.* **37**, 4302–4315 (2017).

74. J. Mao, B. Yan, "Global monthly mean leaf area index climatology, 1981–2015" (ORNL Distributed Active Archive Center (DAAC), Oak Ridge National Laboratory, 2019); https://doi.org/10.3334/ORNLDAAC/1653.

75. S. A. Spawn, H. K. Gibbs, "Global aboveground and belowground biomass carbon density maps for the year 2010" (ORNL Distributed Active Archive Center (DAAC), Oak Ridge National Laboratory, 2020); https://doi.org/10.3334/ORNLDAAC/1763.

76. W. Shangguan, Y. Dai, Q. Duan, B. Liu, H. Yuan, A global soil data set for earth system modeling. *J. Adv. Model. Earth Syst.* **6**, 249–263 (2014).
